# The intervertebral disc contains intrinsic circadian clocks that are regulated by age and cytokines and linked to degeneration

**DOI:** 10.1136/annrheumdis-2016-209428

**Published:** 2017-02-17

**Authors:** Michal Dudek, Nan Yang, Jayalath PD Ruckshanthi, Jack Williams, Elzbieta Borysiewicz, Ping Wang, Antony Adamson, Jian Li, John F Bateman, Michael R White, Raymond P Boot-Handford, Judith A Hoyland, Qing-Jun Meng

**Affiliations:** 1 Faculty of Life Sciences, University of Manchester, Manchester, UK; 2 Wellcome Trust Centre for Cell Matrix Research, University of Manchester, Manchester, UK; 3 Murdoch Children's Research Institute, Parkville, Victoria, Australia; 4 Faculty of Medical and Human Sciences, Centre for Tissue Injury and Repair, University of Manchester, Manchester, UK; 5 NIHR Manchester Musculoskeletal Biomedical Research Unit, Manchester Academic Health Science Centre, Manchester, UK

**Keywords:** Low Back Pain, Cytokines, Arthritis, Chondrocytes

## Abstract

**Objectives:**

The circadian clocks are internal timing mechanisms that drive ∼24-hour rhythms in a tissue-specific manner. Many aspects of the physiology of the intervertebral disc (IVD) show clear diurnal rhythms. However, it is unknown whether IVD tissue contains functional circadian clocks and if so, how their dysregulation is implicated in IVD degeneration.

**Methods:**

Clock gene dynamics in ex vivo IVD explants (from PER2:: luciferase (LUC) reporter mice) and human disc cells (transduced with lentivirus containing *Per2*::luc reporters) were monitored in real time by bioluminescence photon counting and imaging. Temporal gene expression changes were studied by RNAseq and quantitative reverse transcription (qRT)-PCR. IVD pathology was evaluated by histology in a mouse model with tissue-specific deletion of the core clock gene *Bmal1*.

**Results:**

Here we show the existence of the circadian rhythm in mouse IVD tissue and human disc cells. This rhythm is dampened with ageing in mice and can be abolished by treatment with interleukin-1β but not tumour necrosis factor α. Time-series RNAseq revealed 607 genes with 24-hour patterns of expression representing several essential pathways in IVD physiology. Mice with conditional knockout of *Bmal1* in their disc cells demonstrated age-related degeneration of IVDs.

**Conclusions:**

We have established autonomous circadian clocks in mouse and human IVD cells which respond to age and cytokines, and control key pathways involved in the homeostasis of IVDs. Genetic disruption to the mouse IVD molecular clock predisposes to IVD degeneration. These results support the concept that disruptions to circadian rhythms may be a risk factor for degenerative IVD disease and low back pain.

## Introduction

The circadian clocks are internal timing mechanisms which drive ∼24-hour rhythms in physiology and behaviour. In mammals, the central pacemaker suprachiasmatic nuclei (SCN) in the hypothalamus synchronises peripheral clocks in most major body organs.^1–3^ Circadian rhythms coordinate tissue-specific physiology with light/darkness, rest/activity, feeding cycles and body temperature fluctuations.^1^ ^4^ Disruptions to circadian rhythms (during ageing or in shift workers) have been linked to increased risk of diseases (eg, obesity, diabetes, cardiovascular disease and osteoarthritis).^5^ ^6^ At the molecular level, the circadian clock consists of a network of transcriptional activators (*Clock*, *Bmal1*) and repressors (*Per1/2* and *Cry1/2*) organised in a negative feedback loop.^6^ This core oscillator generates 24-hour rhythms in the expression of its core components and a myriad of clock-controlled genes. Depending on the tissue, expression of 3%–16% of the whole transcriptome exhibits a circadian rhythm.^7^

The spine is comprised of bony vertebral bodies alternating with fibrocartilagenous intervertebral discs (IVD). IVD degeneration is among the most prevalent musculoskeletal disorders affecting one in five people under 60 and more than half of the people above 60 years of age.^8^ Low back pain, which is often associated with IVD degeneration, is the number one cause of years lived with disability in the developed countries.^9^ Existing evidence suggests that the IVD is a highly rhythmic tissue, experiencing a diurnal cycle of higher loading (activity phase),^10^ ^11^ followed by a period of low-load recovery (resting phase). Under high load, the pressurised interstitial fluid flows to regions of lower pressure through the outer annulus fibrosus (AF) and the cartilaginous end plate (CEP), resulting in decreased disc height, AF outward bulging and an increase in osmolarity of the central gelatinous nucleus pulposus (NP). During the recovery period, the process is reversed by high osmotic pressure inside the disc causing fluid flow to the NP.^12^ Exchange of nutrients/metabolites that occurs with fluid flow during this cycle maintains disc cell homeostasis.^13^

Consistent with the rhythmic nature of IVD tissue, shift work (a factor known to disrupt circadian rhythms) was reported to be associated with higher risk of low back pain (LBP) and IVD degeneration.^14–18^ We have previously shown that environmental disruption of circadian rhythm in mice, when combined with high fat diet, leads to degeneration of the lumbar IVD tissue in mice.^19^ More recently, changes in the expression of circadian clock genes have been identified in rat IVD tissues following passive smoking (a risk factor for LBP).^20^ However, no studies have examined whether IVD cells express intrinsic circadian clocks, how these IVD clocks are regulated, what their targets are and whether genetic disruption to the IVD clock impact on tissue homeostasis and susceptibility to degeneration.

In this study, we systemically characterised the molecular circadian clock mechanisms in mouse and human IVD tissue/cells. Moreover, by generating a tissue-specific *Bmal1* KO mouse model, our study provides the first genetic evidence linking a core clock factor to IVD degeneration.

## Results

### Intervertebral disc possesses a functional, temperature entrainable circadian clock

**Figure 1:**
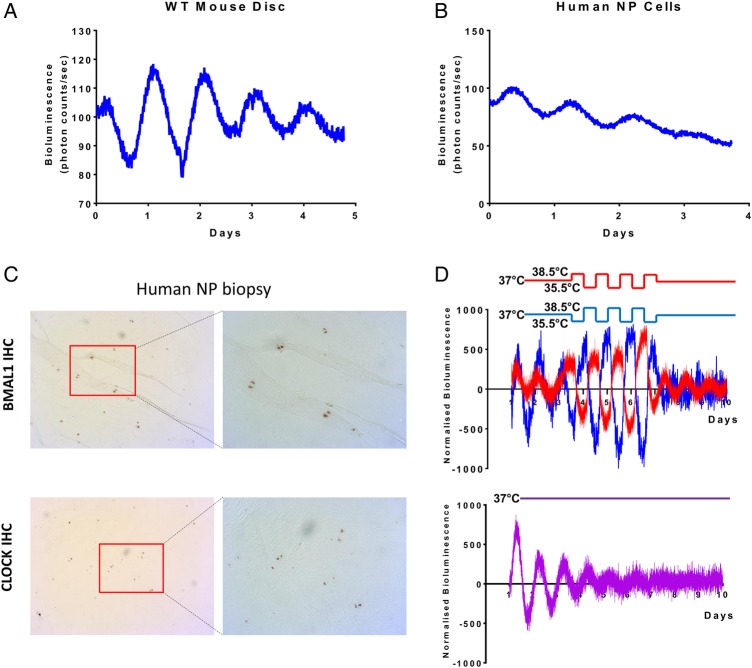
Intervertebral discs (IVDs) possess an autonomous circadian clock. (A) Representative PER2::Luc bioluminescence trace of mouse IVD explant culture (period=23.93±0.247 hours; mean±SD; n=6). (B) Representative trace of human nucleus pulposus (NP) cells transduced with a *Per2::luc* reporter (period=22.52±0.39 hours; mean±SD; n=3). (C) IHC of BMAL1 and CLOCK on NP biopsy of human IVDs (magnification 5× left, 10× right); n=3. (D) Temperature entrainment (n=4). Two IVD explant cultures (represented by red and blue traces) from the same animal were held under antiphase temperature cycles (alternating 12-hour cycles of 38.5°C/35.5°C; baseline temperature=37°C). Third IVD explant culture from the same animal was kept at a constant temperature of 37°C (purple trace below).

To test whether the IVD contains a molecular circadian clock capable of driving circadian rhythm of gene expression, we monitored the dynamics of PER2::Luc protein in IVD explant cultures isolated from PER2::Luc reporter mice.^21^ Real-time bioluminescence photon counting demonstrated robust circadian rhythm of PER2::Luc activity which lasted for more than 5 days, with a period of 23.93±0.10 hours (mean±SEM, n=6, figure 1A). As the IVD comprises two distinct cell types, the NP and AF cells, we wanted to know if both regions exhibit circadian rhythms. Live imaging of the mouse IVD explants using high-sensitivity electron multiplying (EM)-CCD camera revealed rhythmic PER2::Luc signals from both AF and NP cells (see online supplementary videos [Supplementary-material SM2]S1–3). To extend these studies to humans, primary human NP cells were transiently transfected with a vector carrying the luciferase gene under the control of the *Per2* promoter. This approach revealed cell-autonomous circadian oscillations of *Per2::luc* expression, indicating the operation of a functional clock machinery in these human disc cells (figure 1B). Immunohistochemistry (IHC) staining of human NP tissue sections using antibodies against BMAL1 and CLOCK confirmed the presence of these essential circadian clock components in human discs (figure 1C).

10.1136/annrheumdis-2016-209428.supp1 supplementary video

10.1136/annrheumdis-2016-209428.supp2 supplementary video

10.1136/annrheumdis-2016-209428.supp3 supplementary video

One of the key properties of a peripheral circadian clock is their ability to respond to time cues that are controlled by the SCN clock, such as hormones or changes in body temperature. Since the IVDs are not vascularised or innervated (except in pathological conditions),^22^ we hypothesised that daily body temperature oscillations may be a mechanism of clock entrainment for IVDs. To test this, IVD explants from the same mouse were placed in different incubators programmed to have oppositely phased cyclic temperature changes for 4 days (38.5°C for 12 hours/35.5°C for 12 hours, or vice versa), before returning to a constant 37°C. As a control, another IVD explant from the same mouse was incubated under constant 37°C. The PER2::Luc rhythms in IVD explants were all in similar circadian phase for the first 3 days before the temperature protocol (figure 1D). Once the antiphasic protocol was introduced, the oscillations were driven 180° out of phase with each other. Interestingly, the antiphasic oscillations were maintained for at least three more days after the tissues were released to constant temperature. In contrast, the IVD explant that remained at constant temperature gradually lost its ability to oscillate by day 7, mainly due to desynchronisation in culture (figure 1D). These results clearly indicate that temperature cycles that approximate body temperature changes are capable of entraining the circadian phase of the IVD oscillation and enhancing the oscillation amplitude.

### Ageing affects the circadian rhythm of IVDs

**Figure 2:**
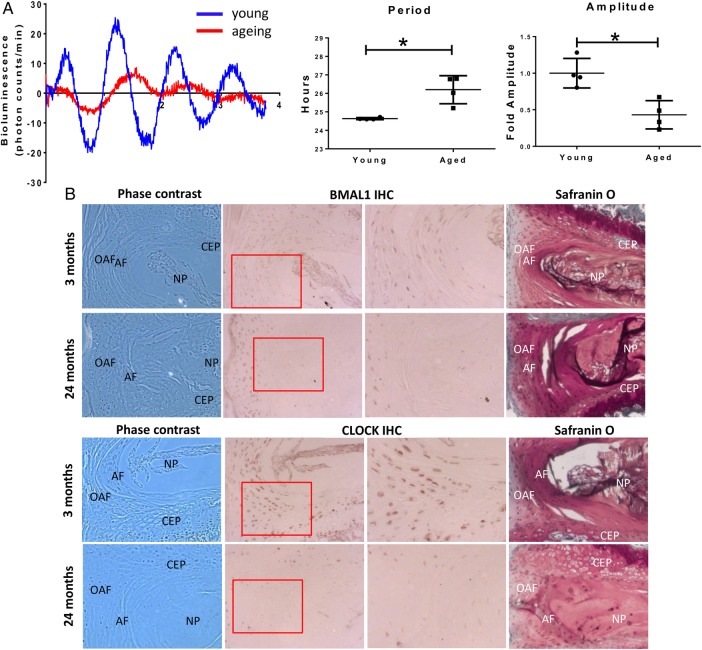
Circadian rhythm of IVD is dampened during ageing. (A) Representative bioluminescence traces of young (2 months) and ageing (12 months) IVDs from PER2::Luc mice. The period was significantly lengthened in older mice (p<0.05) and the amplitude was significantly dampened (p<0.05) (two-tailed non-parametric Mann-Whitney test; n=4); (B) IHC of BMAL1 and CLOCK on young (3 months) and aged (24 months) mouse IVDs; n=4. Magnification 10×. The Safranin O staining panel on the right was included to ease visualisation of the different structures of the IVD. AF, annulus fibrosus; CEP, cartilaginous end plate; IVD, intervertebral disc; NP, nucleus pulposus; OAF, outer annulus fibrosus.

Daily systemic time cues in body temperature and hormone release are known to be altered with ageing.^23^ In addition, intrinsic properties of the clock oscillator could deteriorate with age as well.^23^ ^24^ Indeed, we have previously demonstrated that the amplitude of circadian oscillations in cartilage and tendon tissues dampen with ageing.^25^ ^26^ Therefore, we hypothesised that circadian rhythms may change in ageing disc, compromising the daily control of IVD physiology. To assess this, we compared the oscillations of PER2::Luc expression in mouse IVD explant cultures from animals aged 2 and 12 months (see figure 2A and online supplementary Video S1). The amplitude of oscillations in IVDs from 12 months old mice was severely reduced (by ∼60%) as compared with 2-month-old mice. Additionally, the average period of oscillations was significantly lengthened by 1.6 hours in IVDs from 12-month-old mice (figure 2A). IHC staining showed decreased expression of the core circadian transcription factors BMAL1 and CLOCK in 12-month (see online supplementary figure S1) and 24-month-old mice as compared with 2-month-old mice (figure 2B). These data demonstrate that the IVD clock becomes dysregulated with ageing.

10.1136/annrheumdis-2016-209428.supp4 supplementary figures

### The circadian rhythm of IVD is disrupted by interleukin-1β in a NF-κB-dependent manner

**Figure 3:**
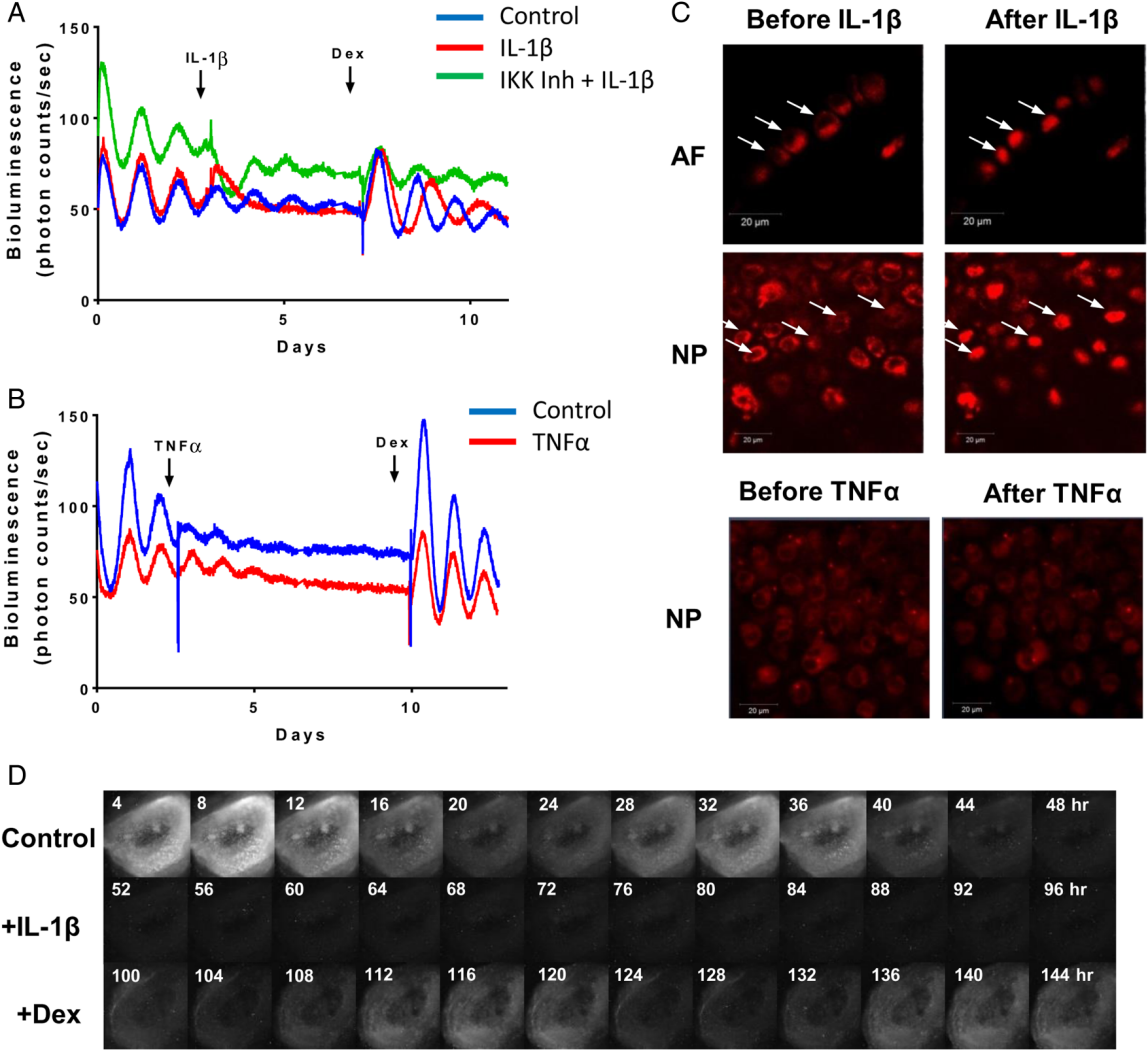
IL1β, but not TNFα, disrupts the circadian rhythm of IVDs. (A) Representative bioluminescence traces of PER2::Luc mouse IVD explants. Arrows indicate time of treatment with IL-1β (5 ng/mL), inhibitor of kappaB kinase (IKK) inhibitor (BMS-345541, 10 µM) and dexamethasone (100 nM). Red trace—treated with IL-1β, green trace—pretreated with IKK inhibitor before addition of IL-1β, blue trace—vehicle control; n=3. (B) Representative bioluminescence traces treated with TNFα (red trace, 40 ng/mL) or control (blue trace). Arrows indicate time of treatments; n=3. (C) Live fluorescence imaging of p65DsRed reporter in mouse IVDs by confocal microscopy before and after treatment with IL-1β or TNFα. Scale bar 20 µm. Arrows indicate the nuclei. (D) Live bioluminescence imaging of an IVD tissue from PER2::Luc mouse, treated with IL-1β (at 48 hours), followed by dexamethasone (at 96 hours). AF, annulus fibrosus; IL, interleukin; IVD, intervertebral disc; NP, nucleus pulposus; TNF, tumour necrosis factor.

Chronic inflammation is a known factor associated with IVD degeneration and lower back pain.^27^ To investigate the effects of catabolic cytokines on disc circadian clock, we treated IVD explants from the PER2::Luc reporter mice with interleukin (IL) 1β, lipopolysaccharide (LPS) and tumour necrosis factor (TNF) α. Tissues were under continuous bioluminescence recording. Treatment with IL-1β (or LPS, see online supplementary figure S2A) resulted in complete disruption of the PER2::Luc circadian rhythm, associated with significant changes of clock genes (*Bmal1, Per2* and *Nr1d1*) (see figure 3A and online supplementary figure S3). The disrupted rhythm could be reinstated by dexamethasone (an anti-inflammatory glucocorticoid, figure 3A) or IL-1RA (an antagonist of IL-1, see online supplementary figure S2B), but not by forskolin (a clock synchronising agent without anti-inflammatory properties, see online supplementary figure S2C). Nuclear factor kappa B (NF-κB) is one of the classical pathways through which IL-1β can mediate its effects. To evaluate the involvement of NF-κB, we used the IKK1/2 inhibitor BMS-345541 to block the activation of NF-κB. The clock-disrupting effect of IL-1β was blocked by pretreating the IVD explant with BMS-345541, supporting a role of NF-κB pathway in the IL-1β-mediated clock disruption. In contrast to IL-1β, treatment of IVD explants with TNFα had no effect on their circadian rhythms (figure 3B). In contrast, both IL-1β and TNFα elicited a strong induction of NF-κB signalling in a lung epithelial cell line, suggesting a possible cell-type-specific response (see online supplementary figure S2D). Next, we took advantage of a transgenic mouse strain expressing the p65-DsRedXP protein fusion construct^28^ to observe the nuclear translocation of p65, one of the major components of the NF-κB complex. Live imaging showed that treatment of IVD explants with IL-1β caused rapid nuclear translocation of p65 both in AF and NP cells. However, addition of TNFα (up to 40 ng/mL) had no effect on p65 translocation (figure 3C).

There are at least two potential mechanisms through which IL-1β could disrupt the IVD circadian rhythm. Individual cells may still have robust clocks but become desynchronised, with their clocks being in different phases, leading to reduced oscillation amplitude, or individual cells may have lost their pacemaking properties. To distinguish between these two possibilities, we used a high-sensitivity EM-CCD camera to visualise the PER2::Luc bioluminescence signals from individual cells in the presence or absence of IL-1β. Consistent with the lack of effect of forskolin, this imaging approach revealed loss of bioluminescence at single cell level, excluding the desynchronisation hypothesis (see figure 3D and online supplementary video S2). Therefore, disruption to the IVD clock could be a hitherto undiscovered response to proinflammatory cytokines.

### Identification of the first IVD circadian transcriptome

**Figure 4:**
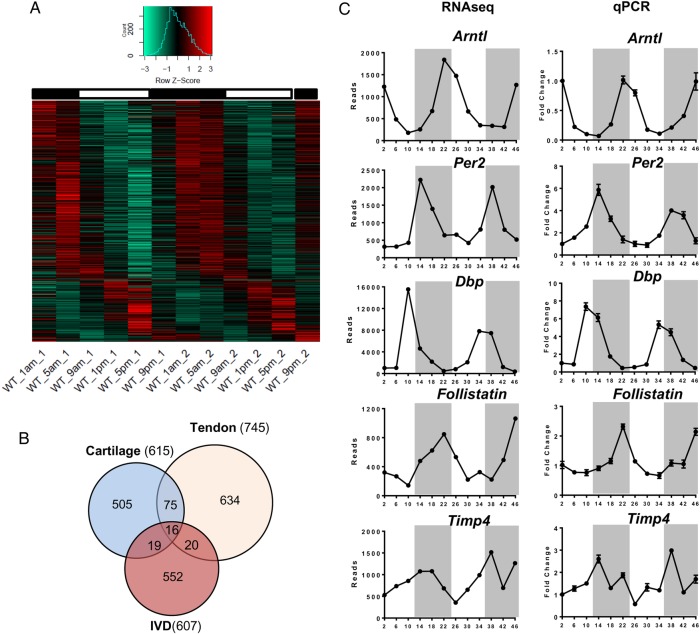
Circadian transcriptome in mouse intervertebral disc (IVD) identified by time-series RNA sequencing. (A) Heat map depicting the expression patterns of the 607 rhythmic genes (3.5% of the IVD transcriptome) identified by JTKCycle. Genes were organised according to timing of peak expression. White bars represent the day; black bars represent the night. (B) Venn diagram comparing the number of rhythmic genes of IVD, cartilage and tendon. (C) qPCR validation of time-dependent expression of clock genes (*Bmal1, Per2* and *Dbp)* and target genes (*Follistatin* and *Timp4*) in mouse IVDs normalised to *Gapdh*. Mean and SEM (n=6). Grey shadow indicates the night phase.

Circadian clocks in different tissues exert their local functions through regulating diverse yet highly tissue-specific set of target genes. To reveal the extent of rhythmic genes in IVD tissue under physiological conditions, we performed a time-series RNAseq study using IVD tissues (collected every 4 hours for 48 hours) from mice kept in 12-hour light/12-hour darkness. We used a well-recognised JTKCycle^29^ algorithm to pick out rhythmic genes. Using P_adjust_<0.05 as a cut-off, we identified 607 genes (3.5% of expressed genes in IVD) with rhythmic 24-hour expression patterns (see figure 4A and online supplementary table S1). Further phase clustering analysis of these rhythmic genes using R package revealed four main clusters (see online supplementary figure S4), with more than 70% of these genes peaking at night time points (representing the active phase of mouse). Gene ontology (GO)-term analysis using topGO revealed dozens of overrepresented functional groups with an adjusted p<0.01, including ‘fatty acid metabolic process’, ‘circadian rhythm’, ‘intracellular protein transmembrane transport’, ‘intrinsic apoptotic signalling pathway’, ‘carboxylic acid metabolic process’ and ‘response to endoplasmic reticulum stress’. We next compared the IVD rhythmic gene list with that of the mouse cartilage and tendon we published earlier.^25^ ^26^ There was a very small number of genes (6%–16%) overlapping between any two of these skeletal tissues, with only 16 genes common to all three, supporting the tissue-specific function of the peripheral clocks (figure 4B). Of these 16 common genes, 8 were core circadian clock genes. The expression profiles of canonical clock genes (*Bmal1*, *Per2, Dbp*) and selected target genes *Follistatin* (a bone morphogenetic proteins (BMP) antagonist)^30^ *and Timp4* (a tissue inhibitor of matrix metalloproteinase (MMPs))^31^ relevant to IVD physiology and catabolism were validated by temporal quantitative reverse transcription (qRT)-PCR in mouse IVD tissues (see figure 4C and online supplementary figure S5).[Supplementary-material SM6]

10.1136/annrheumdis-2016-209428.supp5 supplementary table List of rhythmic IVD genes with a ~24 hr period.

10.1136/annrheumdis-2016-209428.supp6 supplementary methods

### Targeted deletion of *Bmal1* causes age-dependent IVD degeneration

**Figure 5:**
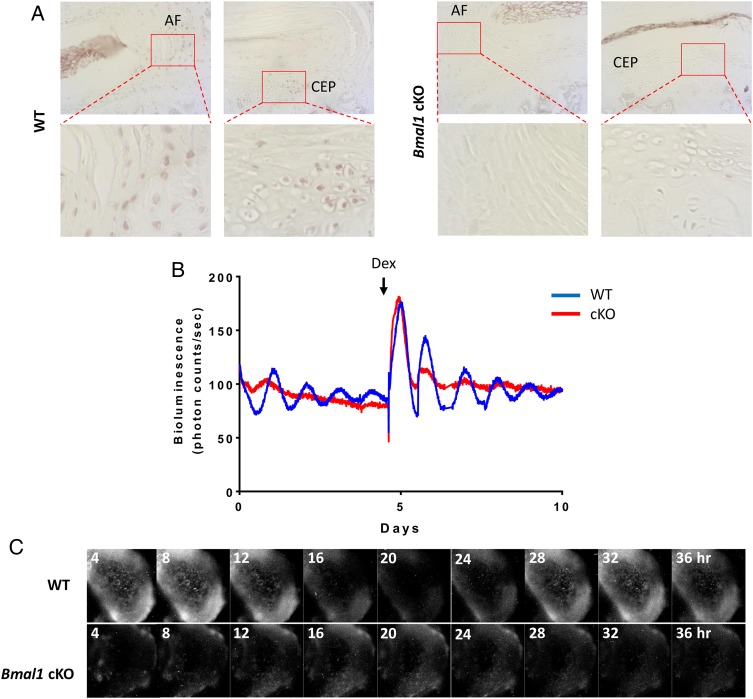
Conditional deletion of *Bmal1* in *Col2a1*-expressing cells results in disruption of the circadian rhythms in mouse intervertebral discs (IVDs). (A) IHC of BMAL1 in 3-month-old wild type (WT) and KO mice (magnification: upper panels 10× and lower panels 40×); n=3. (B) Representative bioluminescence traces of WT (blue) and *Bmal1* cKO (red) mouse IVD explant cultures; n=6. Arrow indicates treatment with dexamethasone. (C) Live bioluminescence imaging of IVDs from WT and *Bmal1* cKO IVDs from mice on a PER2::Luc background.

*Bmal1* is an essential circadian clock component for the generation of 24-hour rhythms. The global *Bmal1* knockout mouse shows multitissue pathologies, including ectopic calcification of IVDs.^32^ However, the severe disruption to whole body circadian rhythms confounds interpretation of phenotype. To evaluate the function of local IVD clocks, we produced a conditional KO mouse model (Col2a1-*Bmal1* KO, conditional knockout (cKO)) with a cell-type-specific abolition of the transcription factor *Bmal1* in α1(II) collagen-expressing cells, including NP and AF cells, and chondrocytes.^33^ We have previously shown that the central SCN clock and behavioural locomotion rhythms in the cKO mice are not affected.^33^ IHC staining of IVDs confirmed loss of BMAL1 expression in the majority of the AF cells and chondrocytes of the CEP in cKO mice (figure 5A). The cKO mouse was crossed with the PER2::Luc mouse to enable real-time tracking of clock rhythms. Photon counting of PER2::Luc bioluminescence demonstrated a lack of circadian oscillations in the cKO IVDs, with no response to dexamethasone treatment (figure 5B). Bioluminescence imaging of the cKO IVDs confirmed lack of circadian oscillations of PER2::Luc in both AF and NP cells (see figure 5C and online supplementary video S3).

**Figure 6:**
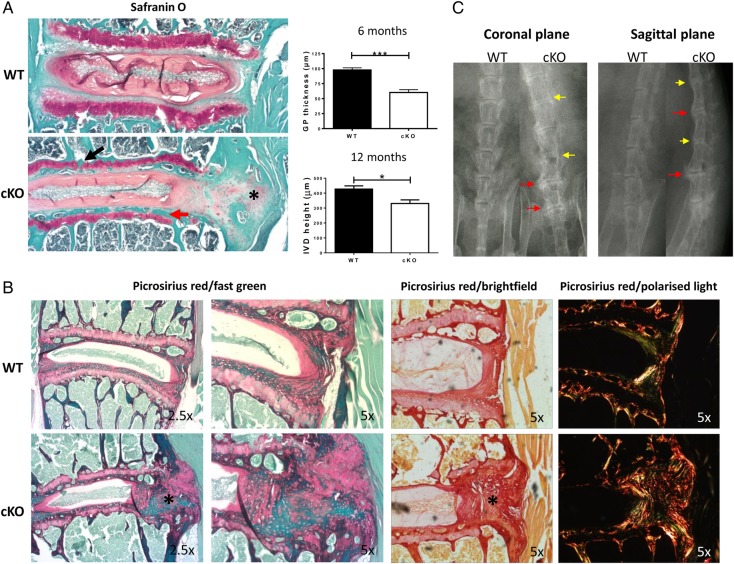
Loss of *Bmal1* leads to degeneration of IVDs and cartilaginous tissues of the spine. (A) Safranin O staining of 12-month-old WT and *Bmal1* cKO mouse lumbar IVDs; n=4. Red arrow—loss of cartilaginous end plate (CEP); black arrow—fragmentation of growth plate; *—fibrosis (magnification 2.5×). Analysis of the intervertebral disc (IVD) height and growth plate thickness was shown (two-tailed non-parametric Mann-Whitney test; n=4) *p<0.05; ***p<0.001. (B) Picrosirius red staining of lumbar IVDs from 12-month-old WT and *Bmal1* cKO mouse showing organisation of collagen (magnification 2.5× left and 5× right panels); n=4. Images were visualised under brightfield or polarised light. (C) X-ray radiography of 12-month-old WT and *Bmal1* cKO mouse spines; n=3. Yellow arrows—calcification of IVDs; red arrows—calcification of tissues surrounding the IVDs.

Histological analysis revealed early signs of degeneration of the lumbar IVDs in cKO mouse at 6 months of age, such as thinning of the growth plate of vertebral body (figure 6A), and gradual disappearance of the CEP (see online supplementary figure S6). At 12 months, there was widespread degeneration of lumbar IVDs in cKOs. Bone bridges appeared within the growth plate, the CEP was almost completely replaced by bone (figure 6A, black arrow) and the height of the disc was significantly reduced in cKO IVDs (figure 6A). In addition, staining with Safranin O and picrosirius red revealed disorganisation of the outer annulus structure and signs of fibrosis (with organised collagen bundles) appearing at the periphery of the IVDs (figure 6A–C, asterisk). Finally, using X-ray studies, the cKO mice showed clear signs of calcification and narrowing of spaces between vertebrae at 6 months (in tail IVDs, data not shown) and 12 months (in lumbar IVDs, figure 6C). No signs of degeneration were evident in age-matched wild type (WT) mice up to the age of 12 months (figure 6B). However, similar degenerative changes to the cKO mutants were visible in WT mice at 24 months of age (see online supplementary figure S7), suggesting the possibility that loss of *Bmal1* and/or circadian rhythm in IVD cells leads to accelerated ageing of the tissue. Terminal deoxynucleotidyl transferase dUTP nick end labeling (TUNEL) assay and qPCR were performed to explore the underlying mechanisms for the observed phenotype. There were no obvious signs of apoptosis, although significant upregulation of catabolism-related genes (*Adamts1, Adamts5, Adamts15* and *Follistatin*) was observed in cKO IVDs (see online supplementary figures S8 and 9). Together, these results indicate the essential role of the locally expressed core clock factor BMAL1 in IVD homeostasis, loss of which led to profound tissue degeneration.

## Discussion

Low back pain is among the most prevalent spinal diseases associated with increasing age, with over 80% of the UK population predicted to experience back pain within their lifetime. Progressive degeneration of the IVD tissue, partly caused by increased catabolism driven by inflammatory/catabolic cytokines, is a major contributing factor in LBP.^34^ It has long been known that the physiology of IVD is under strong influence by a diurnal rhythm associated with the rest/activity cycles, that is, daily cycles of loading (activity phase) and low-load recovery (resting phase).^10–13^ Exchange of nutrients/metabolites that occurs with fluid flow during this cycle maintains disc cell homeostasis. Recent epidemiological and experimental studies have linked shift work (in humans) and chronic disruption of circadian rhythms (in mice) to higher risk of IVD degeneration.^14^ ^15^ ^17–19^ However, our study represents the first critical analysis of the molecular and cellular mechanisms of the IVD clock under physiological and pathological conditions. Using the clock gene reporter mouse/cell models, as well as a conditional *Bmal1* KO mouse model that had disrupted IVD clock, we established autonomous circadian clocks in mouse and human IVD cells that respond to temperature cycles, dampen with age and become dysregulated by catabolic cytokines. Genetic disruption to the mouse IVD molecular clock predisposes to IVD degeneration. Global *Bmal1* KO also showed a phenotype in the skeletal system, including the spine. However, our conditional KO model allows us to conclude the essential role of locally expressed BMAL1 or circadian rhythm in maintaining IVD homeostasis. These results support the notion that disruptions to circadian rhythms during ageing or in shift workers may be a contributing factor for the increased susceptibility to degenerative IVD diseases and low back pain.

We also revealed for the first time the circadian transcriptome of the IVD tissue. Of particular interest are the genes and pathways that have been previously implicated in IVD physiology and pathology, such as genes involved in matrix homeostasis/repair (eg, Follistatin, Timp4, Adamts1, Adamts5, Adamts15 and Adam17),^30^ ^31^ mitochondria function and fatty acid metabolism (eg, Pex1, Pex2, Pex5, Pex15, Adipoq, Adipor2 and Fasn).^35^ ^36^ Although glucose and anaerobic glycolysis represent major metabolic pathways in IVD, there is evidence that mitochondria in the NP are functional and they retain the capacity to metabolise fatty acids through mitochondrial oxidative metabolism.^35^ Other relevant pathways include endoplasmic reticulum (ER) stress and apoptosis (eg, Aifm1, Atf6, Chac1, Bak1, Bbc3, Opa1 and Fas).^37^ ^38^ The diverse clock-controlled pathways identified by this approach implicate circadian rhythm as a critical regulatory mechanism for IVD biology.

Using IVD tissue explants, we have identified the disruption of the circadian clock in IVD as hitherto undiscovered response to proinflammatory cytokines. Similar clock disruptions by inflammatory cytokines have been found in other cell types, such as in macrophages,^39^ synovial fibroblasts^40^ and chondrocytes.^28^ The involvement of NF-κB pathway in mediating the effects of IL-1 is consistent with our earlier findings in chondrocytes, where NF-κB interferes with the core clock complex to disrupt circadian pacemaking.^28^ Given the diverse pathways controlled by the IVD clock, cytokine-mediated circadian disruption may be involved in driving key aspects of the catabolic response of IVD to chronic inflammation. Therefore, there is the possibility of stabilising IVD clock rhythm as a novel strategy to combat tissue catabolism. Although the concentration we used for IL-1β (5 ng/mL) in these tissue explant studies was higher than that in degenerative IVD (∼50 pg/mL), this dose is in line with most ex vivo/in vitro studies. We also identified a lack of response of the IVD clock (and cartilage clock)^28^ to TNFα, possibly due to the defective NF-κB nuclear translocation. These findings suggest that IL-1 and TNFα may act on distinct downstream pathways and regulate different target genes within the IVD, as seen in chondrocytes. In SW1353 chondrocyte-derived cells, catabolic genes such as IL-6, BMP-2, MMP13 and cyclooxygenase (COX)-2 only respond to IL-1, with almost no response to TNFα.^41^ ^42^ Such results are intriguing because we have shown that IL-1β plays a more prominent role in driving disc degeneration than TNFα.^43^ ^44^ Therefore, anti-inflammatory drugs that selectively target IL-1 are more likely to bring therapeutic benefits.

In conclusion, our results provide a firm basis for future studies that aim to elucidate the functional implication and therapeutic potential of the human IVD circadian rhythm in health and disease of the spine.
