# Supplementary material for: The intervertebral disc contains intrinsic circadian clocks that are regulated by age and cytokines and linked to degeneration
Source: Ann Rheum Dis. 2017 Feb 17;76(3):576–84. doi: 10.1136/annrheumdis-2016-209428 (PMC5446006; doi:10.1136/annrheumdis-2016-209428)
Supplement: supplementary figures [file annrheumdis-2016-209428supp002.pdf]

Supplementary Figures

Figure S1. Reduced expression of BMAL1 and CLOCK in ageing IVDs. IHC of BMAL1 and CLOCK on sections of IVDs from 3 and 12 months old mice; n=4. Magnification, 5x left and middle panels, 10x right panels. BMAL1 staining was visible in the AF, but not the CEP, of 12 month old mice. CLOCK staining was largely absent in both AF and CEP in 12 month old mice.

Figure S2. Effects of LPS, IL-1RA, forskolin and TNFα on IVD oscillations. Representative bioluminescence traces of PER2::Luc mouse IVD explants; n=3. (A) LPS treatment (1 µg/mL, red trace) disrupted the rhythm, which could be rescued by treatment with dexamethasone (100 nM). (B, C) Disrupted circadian rhythm by IL-1β treatment (5 ng/mL, red trace) was not rescued by application of forskolin (10 µM), but by pre-treatment with IL-1RA (1 µg/mL) . Arrows indicate time of treatment. (D) Both IL-1β (5 ng/mL) and TNFα (40 ng/mL) induced strong NFkB signalling in lung epithelial cells stably transfected with NFκB::luc reporter. Representative, n=3.

Figure S3. Effects of IL-1β on endogenous clock gene expression. qPCR of several clock genes, *Adamts1* and *IL-6* in IVD explants upon IL-1β treatment (5 ng/mL for 4 hours). *, p<0.05; ***, p<0.001, n=4.

Figure S4. Phase clustering analysis of rhythmic genes in mouse IVDs. Clustering analysis was performed using cluster (A) and Rtsne (B) of R package. These analyses revealed 4 main clusters with different peak times (two at night and two during the day). Example genes for each cluster were highlighted. There was a good concordance between these two methods of analysis.

Figure S5. Time course qPCR of *β-actin* in mouse IVDs. Note the lack of circadian rhythms. Mean and SEM (n = 6).

Figure S6. Early onset of IVD degeneration in IVDs from 6 month old cKO mice. Safranin O/methyl green staining revealed gradual disappearance of CEP in the *Bmal1* cKO mouse (black arrow); n=4.

Figure S7. Spontaneous degeneration of IVDs from aged WT mice. Picrosirius red staining and polarised light microscopy were performed on IVDs from 3 and 24 month old wild type mice; n=4. The aged WT mouse IVDs display a phenotype similar to *Bmal1* cKO IVDs, with fibrosis of the outer AF composed of bundles of organised collagen visible under polarised light microscope.

Figure S8. TUNEL staining of IVDs from 12 months old WT and *Bmal1* cKO mice. Note there were no detectable signs of apoptosis in either WT or cKO IVDs. N=4.

Figure S9. qPCR of catabolic genes in IVDs from 3 months old WT and *Bmal1* cKO mice. Mean and SEM (n = 4). *, p<0.05; **, p<0.01; ***, p<0.001.

Fig S1

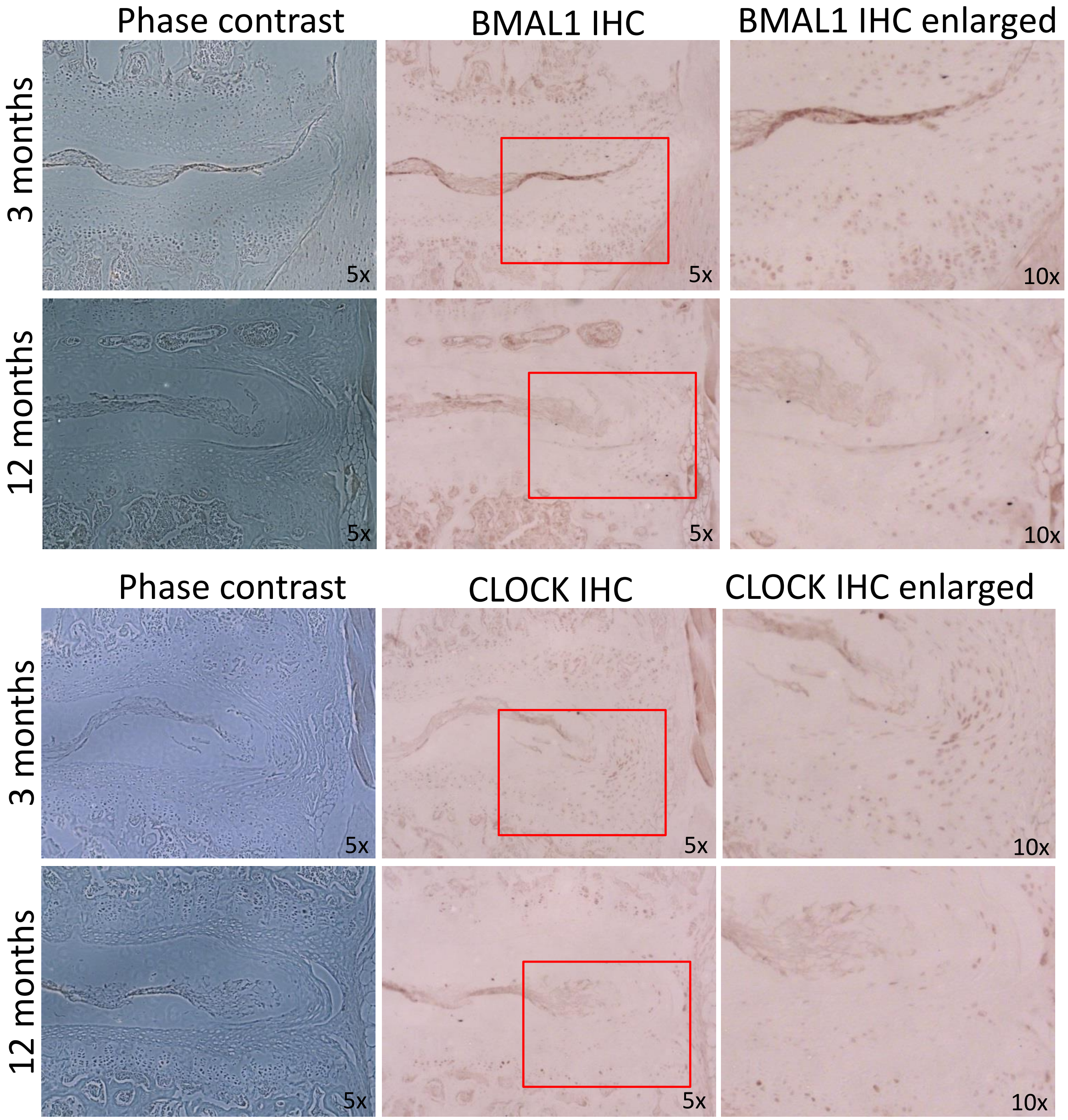
Phase contrast
BMAL1 IHC
BMAL1 IHC enlarged
3 months
12 months
5x
5x
10x
5x
5x
10x
Phase contrast
CLOCK IHC
CLOCK IHC enlarged
3 months
12 months
5x
5x
10x
5x
5x
10x

Fig S2

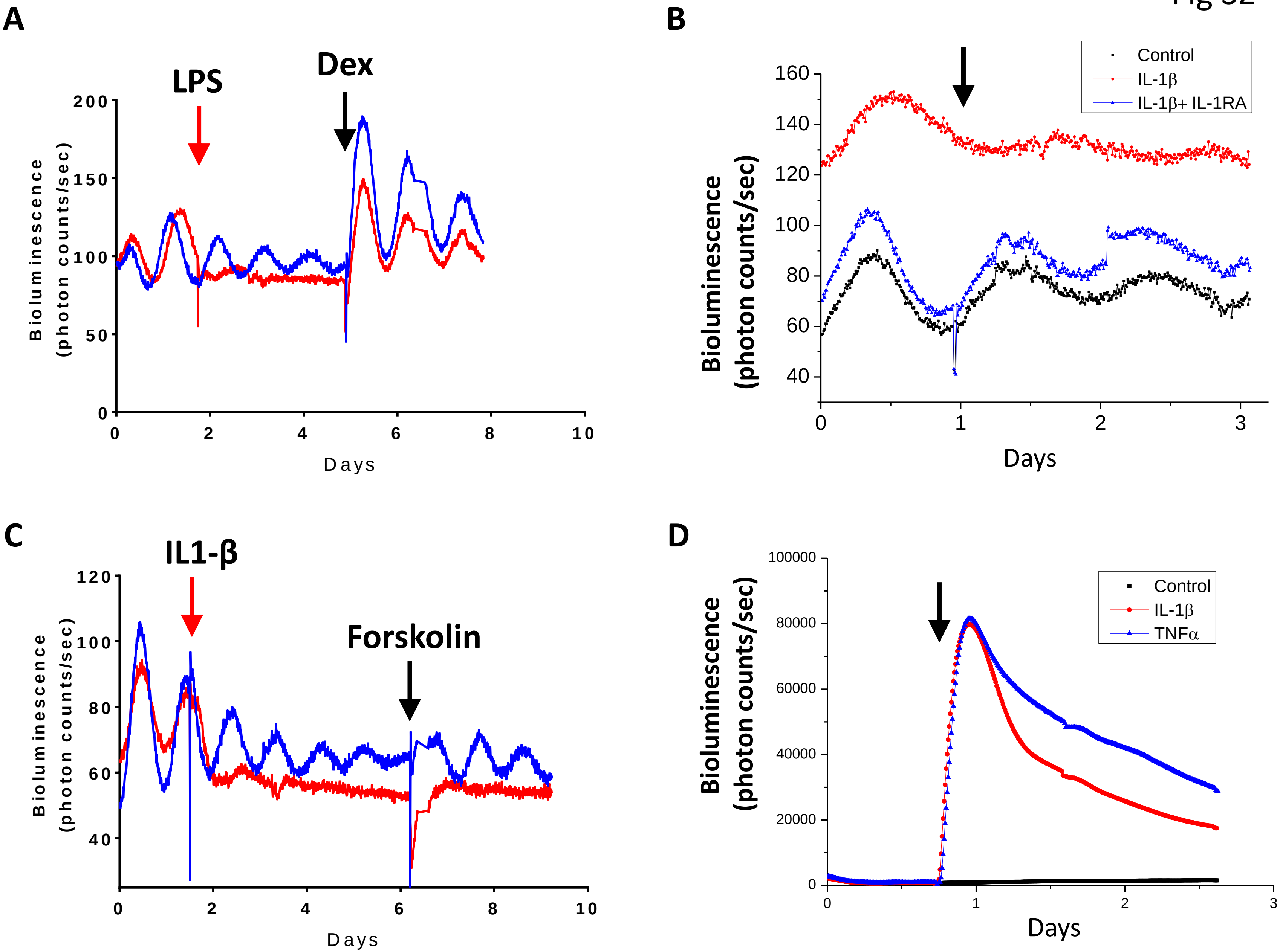
A
LPS
Dex
Bioluminescence
(photon counts/sec)
200
150
100
50
0
0
2
4
6
8
10
Days
B
Control
IL-1β
IL-1β+ IL-1RA
160
140
120
100
80
60
40
0
1
2
3
Bioluminescence
(photon counts/sec)
Days
C
IL1-β
Forskolin
120
100
80
60
40
Bioluminescence
(photon counts/sec)
0
2
4
6
8
10
Days
D
100000
80000
60000
40000
20000
0
Control
IL-1β
TNFα
Bioluminescence
(photon counts/sec)
0
1
2
3
Days

Fig S3

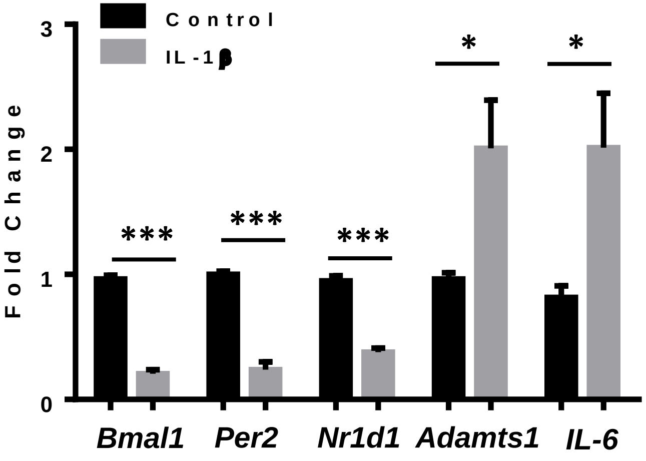

Fig S4

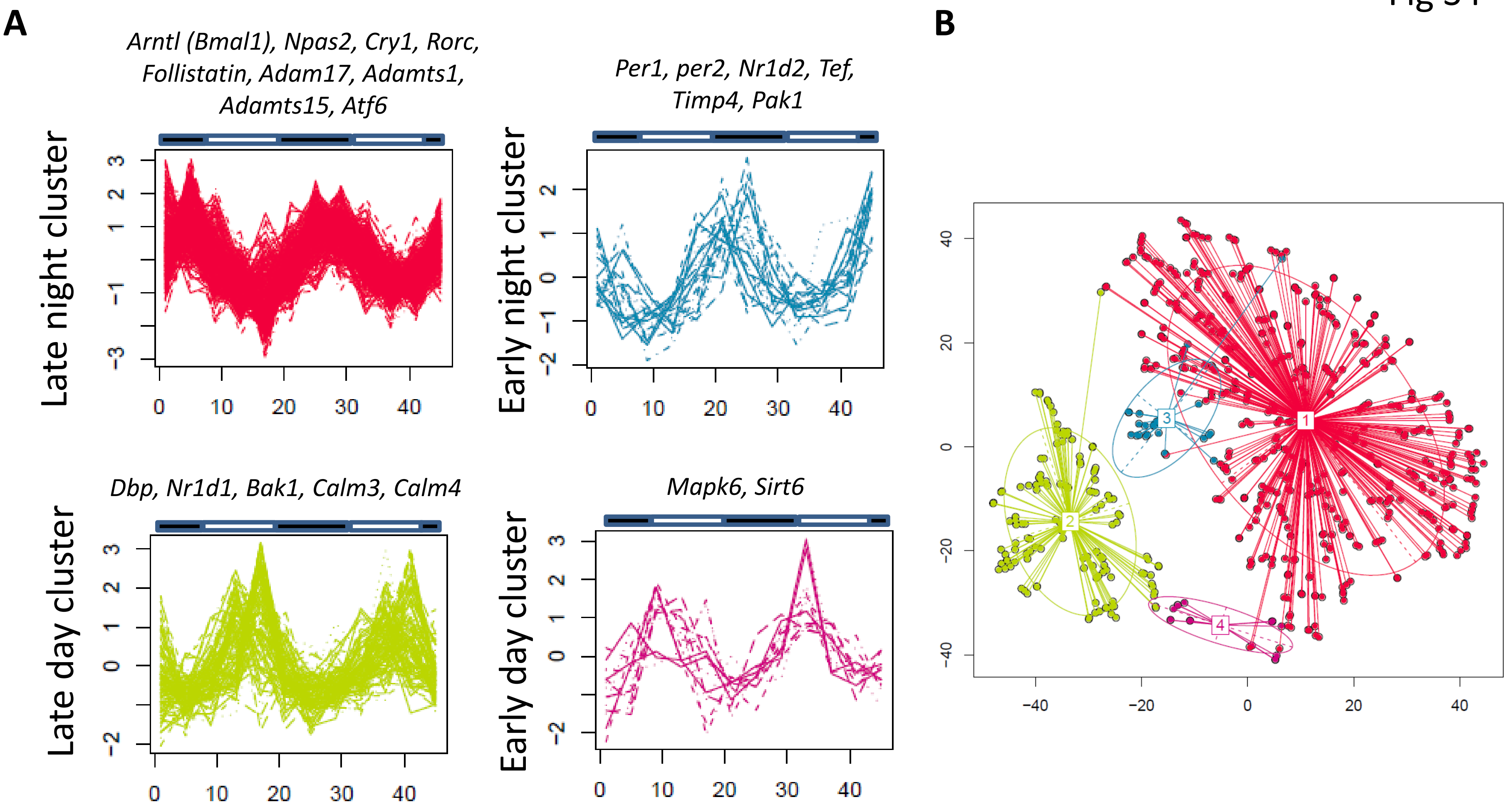
A
Arntl (Bmal1), Npas2, Cry1, Rorc, Follistatin, Adam17, Adamts1, Adamts15, Atf6
Late night cluster
Per1, per2, Nr1d2, Tef, Timp4, Pak1
Early night cluster
Dbp, Nr1d1, Bak1, Calm3, Calm4
Late day cluster
Mapk6, Sirt6
Early day cluster
B
1
2
3
4

Fig S5

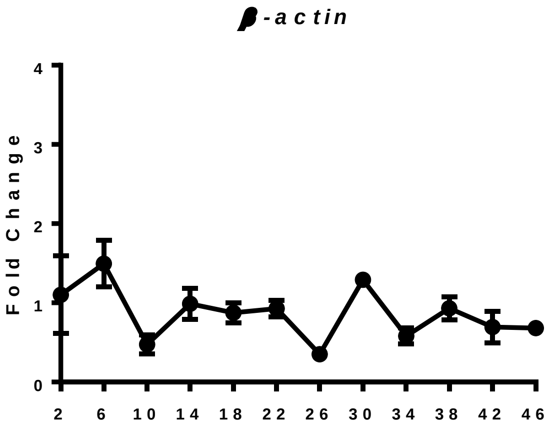

Circadian time, hrs

Fig S6

6months

WT cKO

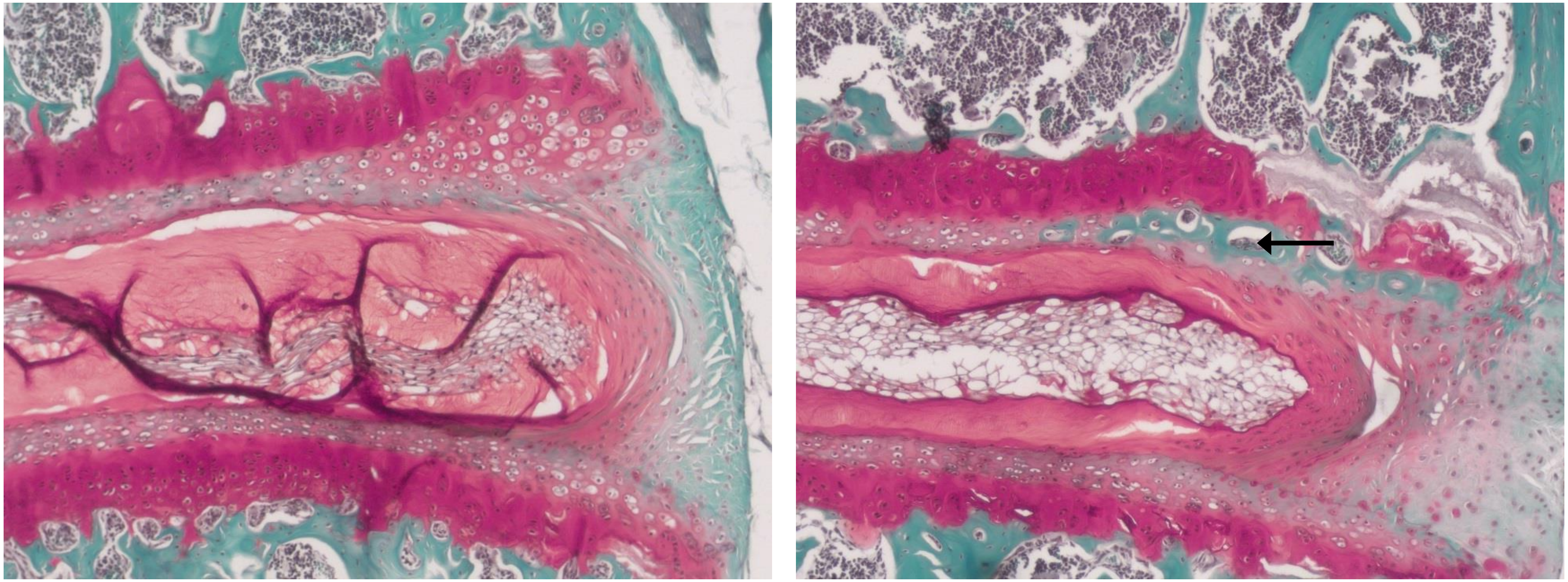

Fig S7

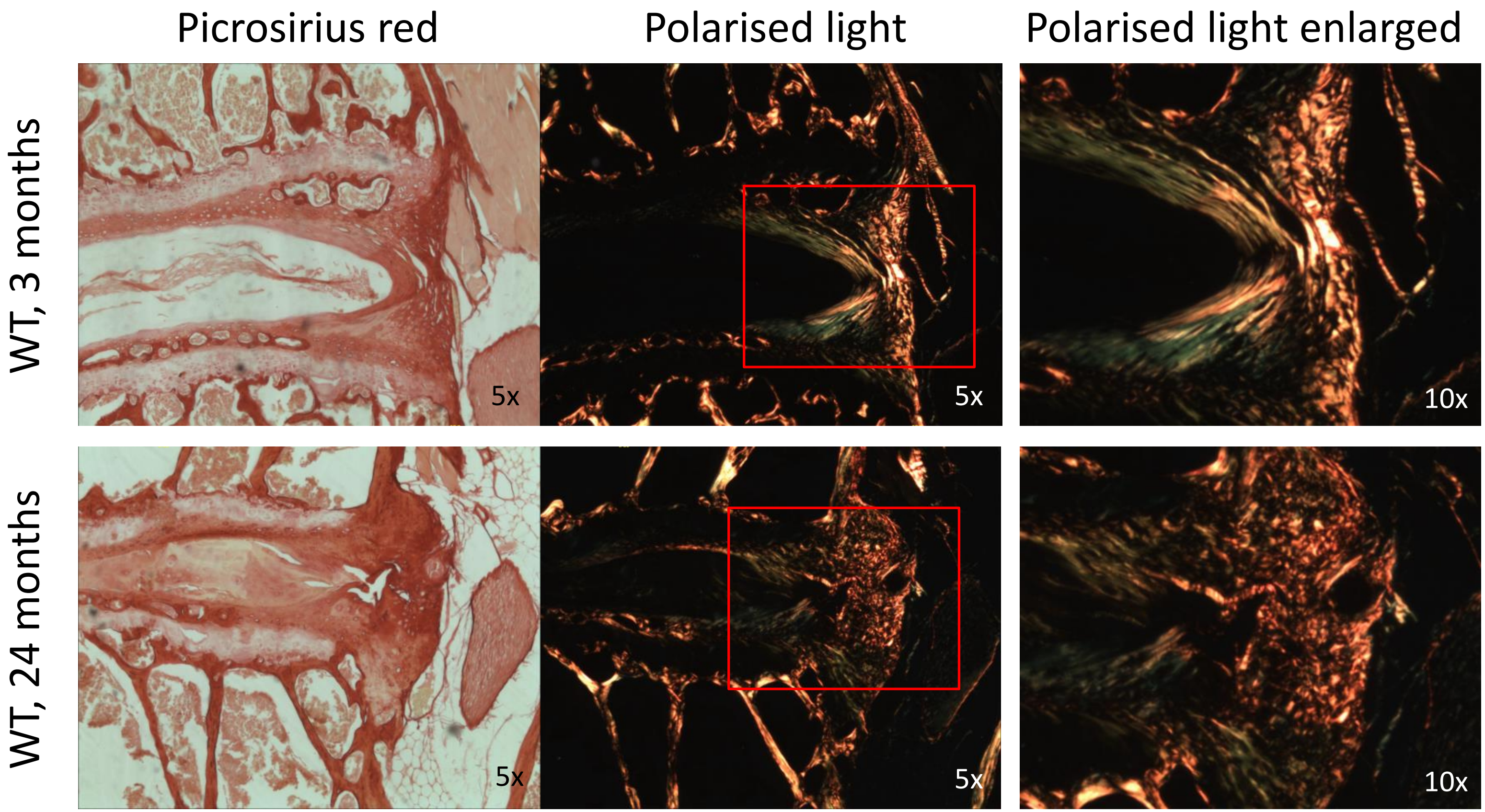
Picrosirius red
Polarised light
Polarised light enlarged
WT, 3 months
WT, 24 months
5x
5x
10x
5x
5x
10x

Fig S8

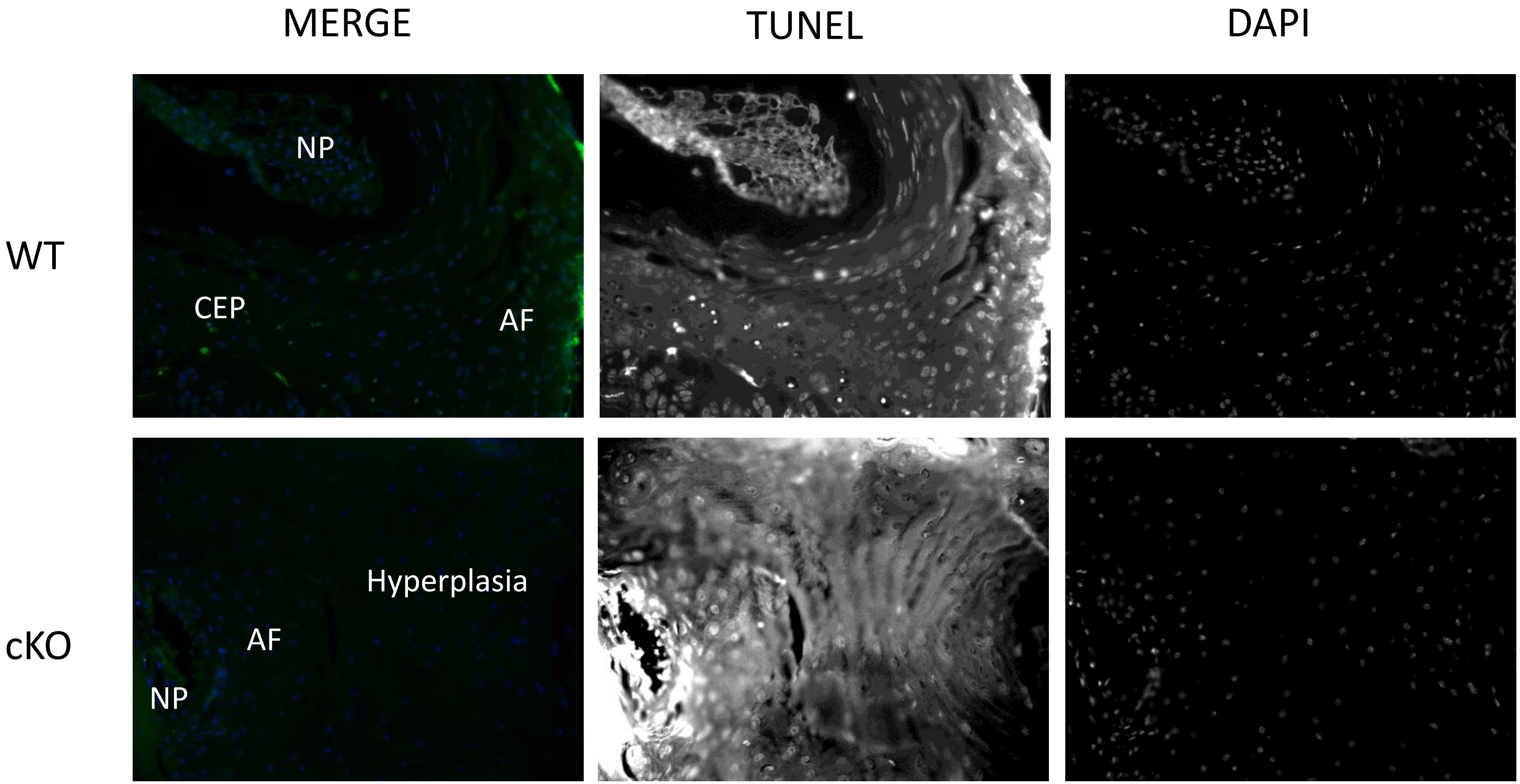

Fig S9

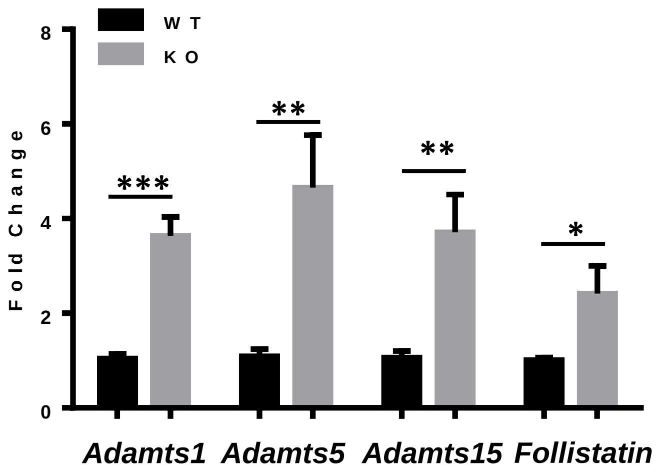
WT
KO
Fold Change
0
2
4
6
8
***
**
**
*
Adamts1
Adamts5
Adamts15
Follistatin
