# Supplementary material for: The intervertebral disc contains intrinsic circadian clocks that are regulated by age and cytokines and linked to degeneration
Source: Ann Rheum Dis. 2017 Feb 17;76(3):576–84. doi: 10.1136/annrheumdis-2016-209428 (PMC5446006; doi:10.1136/annrheumdis-2016-209428)
Supplement: supplementary table — List of rhythmic IVD genes with a ~24 hr period. [file annrheumdis-2016-209428supp003.pdf]

Supplementary Table 1. List of rhythmic IVD genes with a ~24 hr period.

ID	gene_name	ADJ.P	PERIOD	LAG
ENSMUSG00000020917.11	Acly	8.79E-06	24	4
ENSMUSG00000038084.10	Opa1	9.81E-05	24	4
ENSMUSG00000020283.5	Pex13	9.81E-05	24	4
ENSMUSG00000009687.8	Fxyd5	0.000265	24	12
ENSMUSG00000027086.10	Fastkd1	0.000265	24	0
ENSMUSG00000046417.8	Fam211a	0.000265	24	2
ENSMUSG00000022053.7	Ebf2	0.000265	24	5
ENSMUSG00000047797.8	Gjb1	0.000265	24	16
ENSMUSG00000028989.3	Angptl7	0.000645	24	20
ENSMUSG00000025809.9	Itgb1	0.000645	24	4
ENSMUSG00000074766.4	Ism1	0.000645	24	3
ENSMUSG00000026643.10	Nmt2	0.000645	24	4
ENSMUSG00000019883.4	Echdc1	0.000645	24	4
ENSMUSG00000095512.1	Gm17222	0.000645	24	4
ENSMUSG00000047370.4	Gm7367	0.000645	24	12
ENSMUSG00000040605.6	Bace2	0.000645	24	4
ENSMUSG00000039956.2	Mrap	0.000645	24	4
ENSMUSG00000078234.5	Klhdc7a	0.000645	24	4
ENSMUSG00000073176.4	Zfp449	0.000645	24	4
ENSMUSG00000090305.1	Gm5459	0.000645	24	16
ENSMUSG00000029054.2	Gabrd	0.000645	24	2
ENSMUSG00000030972.5	Acsm5	0.001042	24	4
ENSMUSG00000021903.5	Galnt15	0.00144	24	20
ENSMUSG00000041607.10	Mbp	0.00144	24	18
ENSMUSG00000032010.8	Usp2	0.00144	24	22
ENSMUSG00000059824.4	Dbp	0.00144	24	18
ENSMUSG00000026922.7	Agpat2	0.00144	24	4
ENSMUSG00000041653.4	Pnpla3	0.00144	24	4
ENSMUSG00000026663.6	Atf6	0.00144	24	4
ENSMUSG00000029482.3	Aacs	0.00144	24	4
ENSMUSG00000090084.1	Srpx	0.00144	24	4
ENSMUSG00000039364.7	Sectm1b	0.00144	24	16
ENSMUSG00000051367.8	Six1	0.00144	24	6
ENSMUSG00000040370.7	Lyrm5	0.00144	24	2
ENSMUSG00000001700.9	Gramd3	0.00144	24	4
ENSMUSG00000031959.8	Wdr59	0.00144	24	2
ENSMUSG00000044645.7	Gm7334	0.00144	24	2
ENSMUSG00000025940.6	Tmem70	0.00144	24	4
ENSMUSG00000036225.8	Kctd1	0.00144	24	4
ENSMUSG00000021418.9	Rpp40	0.00144	24	2
ENSMUSG00000027663.6	Zmat3	0.00144	24	4
ENSMUSG00000031725.8	Ces1f	0.00144	24	4
ENSMUSG00000013523.7	Bcas1	0.00144	24	16
ENSMUSG00000073233.3	Gm9144	0.00144	24	16
ENSMUSG00000037016.7	Frem2	0.00144	24	4
ENSMUSG00000047473.8	Zfp30	0.00144	24	2
ENSMUSG00000025584.11	Pde8a	0.00144	24	4
ENSMUSG00000086432.1	B430119L08Rik	0.00144	24	6
ENSMUSG00000079168.3	Cd209g	0.00144	24	4

ENSMUSG00000074715.2	Ccl28	0.00144	24	4
ENSMUSG00000087235.1	Gm4750	0.00144	24	16
ENSMUSG00000044951.7	Mylk4	0.002993	24	22
ENSMUSG00000082476.1	Gm12242	0.002993	24	15
ENSMUSG00000076432.6	Ywhaq	0.002993	24	4
ENSMUSG00000063427.7	Rps10-ps2	0.002993	24	16
ENSMUSG00000040181.8	Fmo1	0.002993	24	2
ENSMUSG00000008540.5	Mgst1	0.002993	24	4
ENSMUSG00000021877.5	Arf4	0.002993	24	4
ENSMUSG00000029190.13	D5Ertd579e	0.002993	24	2
ENSMUSG00000036078.10	Sigmar1	0.002993	24	4
ENSMUSG00000022774.6	Ncbp2	0.002993	24	2
ENSMUSG00000027309.12	4930402H24Rik	0.002993	24	4
ENSMUSG00000022707.10	Gbe1	0.002993	24	4
ENSMUSG00000020777.10	Acox1	0.002993	24	2
ENSMUSG00000023832.7	Acat2	0.002993	24	3
ENSMUSG00000028567.8	Txndc12	0.002993	24	4
ENSMUSG00000023022.7	Lima1	0.002993	24	3
ENSMUSG00000039377.6	Hlx	0.002993	24	12
ENSMUSG00000041737.7	Tmem45b	0.002993	24	4
ENSMUSG00000020932.8	Gfap	0.002993	24	16
ENSMUSG00000021765.7	Fst	0.002993	24	4
ENSMUSG00000029208.10	Guf1	0.002993	24	2
ENSMUSG00000026749.5	Nek6	0.002993	24	2
ENSMUSG00000038517.9	Tbkbp1	0.002993	24	14
ENSMUSG00000005501.8	Usp40	0.002993	24	1
ENSMUSG00000020829.9	Slc46a1	0.002993	24	8
ENSMUSG00000094686.1	Ccl21a	0.002993	24	6
ENSMUSG00000073633.3	Fbxo36	0.002993	24	5
ENSMUSG00000027684.10	Mecom	0.002993	24	4
ENSMUSG00000052593.9	Adam17	0.002993	24	4
ENSMUSG00000040502.5	Mar-09	0.002993	24	2
ENSMUSG00000090659.2	Zfp493	0.002993	24	1
ENSMUSG00000040717.5	Il17rd	0.002993	24	4
ENSMUSG00000027792.5	Bche	0.002993	24	4
ENSMUSG00000054000.4	Tusc1	0.002993	24	14
ENSMUSG00000033182.6	Kbtbd12	0.002993	24	4
ENSMUSG00000037577.6	Ephx3	0.002993	24	4
ENSMUSG00000032517.9	Mobp	0.002993	24	16
ENSMUSG00000092746.2	Gm22179	0.002993	24	6
ENSMUSG00000028572.7	Hook1	0.002993	24	18
ENSMUSG00000029503.10	P2rx2	0.002993	24	16
ENSMUSG00000019890.3	Nts	0.00442	24	4
ENSMUSG00000029195.7	Klb	0.00442	24	4
ENSMUSG00000039099.7	Wdr93	0.00442	24	6
ENSMUSG00000037071.2	Scd1	0.005847	24	4
ENSMUSG00000025153.8	Fasn	0.005847	24	4
ENSMUSG00000021775.10	Nr1d2	0.005847	24	19
ENSMUSG00000030082.8	Sec61a1	0.005847	24	4
ENSMUSG00000021610.7	Clptm1l	0.005847	24	4

ENSMUSG00000022878.5	Adipoq	0.005847	24	4
ENSMUSG00000024286.7	Ccny	0.005847	24	2
ENSMUSG00000037470.8	Uggt1	0.005847	24	4
ENSMUSG00000009563.10	Tor2a	0.005847	24	4
ENSMUSG00000025007.7	Aldh18a1	0.005847	24	6
ENSMUSG00000022351.8	Sqle	0.005847	24	2
ENSMUSG00000028343.4	Erp44	0.005847	24	4
ENSMUSG00000060450.7	Rnf14	0.005847	24	2
ENSMUSG00000055116.7	Arntl	0.005847	24	6
ENSMUSG00000056973.6	Ces1d	0.005847	24	2
ENSMUSG00000047539.4	Fbxo28	0.005847	24	2
ENSMUSG00000028496.11	Mllt3	0.005847	24	2
ENSMUSG00000025981.7	Coq10b	0.005847	24	22
ENSMUSG00000069125.1	Rps24-ps2	0.005847	24	16
ENSMUSG00000031489.8	Adrb3	0.005847	24	4
ENSMUSG00000032724.5	Abtb2	0.005847	24	4
ENSMUSG00000001467.4	Cyp51	0.005847	24	3
ENSMUSG00000040564.7	Apoc1	0.005847	24	4
ENSMUSG00000003809.8	Gcdh	0.005847	24	2
ENSMUSG00000022131.2	Gpr180	0.005847	24	2
ENSMUSG00000004394.6	Tmed4	0.005847	24	2
ENSMUSG00000021666.10	Gfm2	0.005847	24	0
ENSMUSG00000041153.3	Osgin2	0.005847	24	2
ENSMUSG00000096795.1	Zfp433	0.005847	24	4
ENSMUSG00000015852.7	Fcrls	0.005847	24	4
ENSMUSG00000026181.11	Ppm1f	0.005847	24	4
ENSMUSG00000005069.6	Pex5	0.005847	24	0
ENSMUSG00000027341.4	Tmem230	0.005847	24	4
ENSMUSG00000030787.3	Lyve1	0.005847	24	2
ENSMUSG00000031358.11	Msl3	0.005847	24	4
ENSMUSG00000089682.3	Bcl2l2	0.005847	24	2
ENSMUSG00000029348.7	Asphd2	0.005847	24	4
ENSMUSG00000038180.5	Spag4	0.005847	24	5
ENSMUSG00000025102.8	3110040N11Rik	0.005847	24	2
ENSMUSG00000020038.9	Cry1	0.005847	24	4
ENSMUSG00000062480.5	Acat3	0.005847	24	4
ENSMUSG00000078923.4	Ube2v1	0.005847	24	14
ENSMUSG00000022074.5	Tnfrsf10b	0.005847	24	6
ENSMUSG00000027938.5	Creb3l4	0.005847	24	4
ENSMUSG00000097643.1	A130051J06Rik	0.005847	24	6
ENSMUSG00000024334.8	H2-Oa	0.005847	24	14
ENSMUSG00000096935.1	1700113A16Rik	0.005847	24	4
ENSMUSG00000002578.9	Ikzf4	0.005847	24	8
ENSMUSG00000034460.8	Six4	0.005847	24	4
ENSMUSG00000031910.8	Has3	0.005847	24	6
ENSMUSG00000040177.9	2310057M21Rik	0.005847	24	4
ENSMUSG00000005268.14	Prlr	0.005847	24	5
ENSMUSG00000073427.3	Gm4924	0.005847	24	2
ENSMUSG00000095969.1	Rnu1a1	0.005847	24	10
ENSMUSG00000074403.2	Hist2h3b	0.005847	24	11

ENSMUSG00000043687.9	1190005I06Rik	0.005847	24	4
ENSMUSG00000069208.5	Zfp825	0.005847	24	4
ENSMUSG00000083320.1	Gm13935	0.005847	24	20
ENSMUSG00000053038.7	Gm6180	0.005847	24	16
ENSMUSG00000044916.4	1700029I15Rik	0.005847	24	4
ENSMUSG00000081497.1	Gm15560	0.005847	24	16
ENSMUSG00000041930.7	Fam222a	0.005847	24	4
ENSMUSG00000097855.1	A930007I19Rik	0.008333	24	6
ENSMUSG00000040165.7	Cd209c	0.008333	24	4
ENSMUSG00000027762.5	Sucnr1	0.008333	24	4
ENSMUSG00000018865.8	Sult4a1	0.008333	24	14
ENSMUSG00000070436.5	Serpinh1	0.01082	24	4
ENSMUSG00000020585.4	Laptm4a	0.01082	24	4
ENSMUSG00000020889.11	Nr1d1	0.01082	24	16
ENSMUSG00000033022.7	Cdo1	0.01082	24	4
ENSMUSG00000024914.10	Drap1	0.01082	24	16
ENSMUSG00000032096.9	Arcn1	0.01082	24	4
ENSMUSG00000020532.12	Acaca	0.01082	24	4
ENSMUSG00000031701.5	Dnaja2	0.01082	24	0
ENSMUSG00000024538.7	Ppic	0.01082	24	4
ENSMUSG00000001494.6	Sost	0.01082	24	14
ENSMUSG00000046711.9	Hmga1	0.01082	24	14
ENSMUSG00000066632.2	Pgk1-rs7	0.01082	24	0
ENSMUSG00000032193.8	Ldlr	0.01082	24	3
ENSMUSG00000027879.3	Sec22b	0.01082	24	2
ENSMUSG00000061838.5	Suclg2	0.01082	24	0
ENSMUSG00000037379.4	Spon2	0.01082	24	7
ENSMUSG00000041891.9	Lman1	0.01082	24	4
ENSMUSG00000023827.2	Agpat4	0.01082	24	6
ENSMUSG00000029231.9	Pdgfra	0.01082	24	4
ENSMUSG00000019872.7	Smpdl3a	0.01082	24	4
ENSMUSG00000027088.4	Phospho2	0.01082	24	2
ENSMUSG00000031467.4	Agpat5	0.01082	24	2
ENSMUSG00000027742.8	Cog6	0.01082	24	2
ENSMUSG00000029009.11	Mthfr	0.01082	24	4
ENSMUSG00000063275.9	Ptpla	0.01082	24	4
ENSMUSG00000029685.9	Asb15	0.01082	24	22
ENSMUSG00000020471.5	Pold2	0.01082	24	13
ENSMUSG00000038416.8	Cdc16	0.01082	24	2
ENSMUSG00000035770.7	Dync1li2	0.01082	24	2
ENSMUSG00000029782.12	Tmem209	0.01082	24	16
ENSMUSG00000021916.9	Glt8d1	0.01082	24	3
ENSMUSG00000028955.3	Vamp3	0.01082	24	2
ENSMUSG00000024014.6	Pim1	0.01082	24	14
ENSMUSG00000034981.9	Parm1	0.01082	24	4
ENSMUSG00000026082.6	Rev1	0.01082	24	22
ENSMUSG00000031610.3	Scrg1	0.01082	24	2
ENSMUSG00000069631.8	Strada	0.01082	24	12
ENSMUSG00000027465.7	Tbc1d20	0.01082	24	4
ENSMUSG00000024150.5	Mcfd2	0.01082	24	2

ENSMUSG00000033453.7	Adamts15	0.01082	24	4
ENSMUSG00000024810.10	Il33	0.01082	24	2
ENSMUSG00000030545.8	Pex11a	0.01082	24	2
ENSMUSG00000031938.8	4931406C07Rik	0.01082	24	2
ENSMUSG00000021559.7	Dapk1	0.01082	24	4
ENSMUSG00000075486.3	Commd6	0.01082	24	3
ENSMUSG00000012422.8	Tmem167	0.01082	24	4
ENSMUSG00000074182.4	Znhit6	0.01082	24	22
ENSMUSG00000047554.7	Tmem41b	0.01082	24	4
ENSMUSG00000028293.8	Slc35a1	0.01082	24	4
ENSMUSG00000007777.3	0610009B22Rik	0.01082	24	5
ENSMUSG00000028256.10	Odf2l	0.01082	24	0
ENSMUSG00000075054.4	Yae1d1	0.01082	24	3
ENSMUSG00000039706.7	Ldb2	0.01082	24	5
ENSMUSG00000032018.7	Sc5d	0.01082	24	3
ENSMUSG00000034413.8	Neurl1b	0.01082	24	4
ENSMUSG00000019791.5	Hint3	0.01082	24	2
ENSMUSG00000097649.1	Gm10561	0.01082	24	4
ENSMUSG00000027555.8	Car13	0.01082	24	4
ENSMUSG00000046636.4	Gm7729	0.01082	24	16
ENSMUSG00000028990.7	Lzic	0.01082	24	2
ENSMUSG00000021567.8	Nkd2	0.01082	24	2
ENSMUSG00000070572.6	Trmt112-ps2	0.01082	24	14
ENSMUSG00000013646.11	Sh3bp5l	0.01082	24	1
ENSMUSG00000063439.6	B9d2	0.01082	24	16
ENSMUSG00000055917.8	Zfp277	0.01082	24	4
ENSMUSG00000039354.10	Smarcal1	0.01082	24	2
ENSMUSG00000060438.4	Rps10-ps1	0.01082	24	16
ENSMUSG00000089797.1	Gm16118	0.01082	24	18
ENSMUSG00000032346.7	Ooep	0.01082	24	4
ENSMUSG00000042340.5	Ctf1	0.01082	24	2
ENSMUSG00000073478.5	D730003I15Rik	0.01082	24	4
ENSMUSG00000020000.7	Moxd1	0.01082	24	4
ENSMUSG00000040321.3	Zfp770	0.01082	24	4
ENSMUSG00000043913.8	Ccdc60	0.01082	24	8
ENSMUSG00000096544.1	Gm4617	0.01082	24	15
ENSMUSG00000082530.1	Gm12168	0.01082	24	16
ENSMUSG00000030336.8	Cd27	0.01082	24	14
ENSMUSG00000015437.4	Gzmb	0.01082	24	13
ENSMUSG00000065824.1	Gm26315	0.01082	24	9
ENSMUSG00000046733.7	Gprc5a	0.01082	24	6
ENSMUSG00000041617.4	Ccdc74a	0.01082	24	8
ENSMUSG00000016181.4	Diexf	0.01082	24	4
ENSMUSG00000091803.1	Cox16	0.01082	24	2
ENSMUSG00000020407.7	Upp1	0.01082	24	6
ENSMUSG00000055137.6	5033411D12Rik	0.01082	24	4
ENSMUSG00000079343.3	Gm5077	0.01082	24	2
ENSMUSG00000037940.9	Inpp4b	0.01082	24	4
ENSMUSG00000039865.7	Slc44a3	0.01082	24	4
ENSMUSG00000039278.10	Pcsk1n	0.01082	24	16

ENSMUSG00000096862.1	Gm13301	0.01082	24	1
ENSMUSG00000079484.6	Phyhd1	0.01082	24	0
ENSMUSG00000097828.1	6430562O15Rik	0.01082	24	4
ENSMUSG00000025475.11	Gpr123	0.01082	24	7
ENSMUSG00000082088.1	Gm15753	0.01082	24	16
ENSMUSG00000086869.2	Gm7809	0.01082	24	0
ENSMUSG00000083567.2	Gm11451	0.01082	24	4
ENSMUSG00000041878.3	8430432A02Rik	0.01082	24	4
ENSMUSG00000074345.3	Tnfaip8l3	0.01082	24	4
ENSMUSG00000033615.9	Cplx1	0.01082	24	16
ENSMUSG00000048186.8	Bend7	0.01082	24	4
ENSMUSG00000081194.1	Gm8424	0.01082	24	4
ENSMUSG00000066438.6	Plekhd1	0.01082	24	16
ENSMUSG00000070577.5	Gm572	0.014955	24	4
ENSMUSG00000017300.9	Tnnc2	0.01909	24	18
ENSMUSG00000028618.5	Tmem59	0.01909	24	2
ENSMUSG00000035493.9	Tgfbi	0.01909	24	2
ENSMUSG00000036309.8	Skp1a	0.01909	24	1
ENSMUSG00000026509.10	Capn2	0.01909	24	2
ENSMUSG00000022816.5	Fstl1	0.01909	24	3
ENSMUSG00000079017.3	Ifi27l2a	0.01909	24	2
ENSMUSG00000008575.11	Nfib	0.01909	24	1
ENSMUSG00000030168.7	Adipor2	0.01909	24	4
ENSMUSG00000027605.12	Acss2	0.01909	24	4
ENSMUSG00000030774.7	Pak1	0.01909	24	20
ENSMUSG00000052837.5	Junb	0.01909	24	13
ENSMUSG00000019370.10	Calm3	0.01909	24	16
ENSMUSG00000022048.8	Dpysl2	0.01909	24	2
ENSMUSG00000027668.7	Mfn1	0.01909	24	0
ENSMUSG00000020893.11	Per1	0.01909	24	18
ENSMUSG00000030062.6	Rpn1	0.01909	24	0
ENSMUSG00000020817.10	Rabep1	0.01909	24	2
ENSMUSG00000022940.10	Pigp	0.01909	24	1
ENSMUSG00000020523.8	Fam114a2	0.01909	24	2
ENSMUSG00000042688.10	Mapk6	0.01909	24	9
ENSMUSG00000029390.7	Tmed2	0.01909	24	4
ENSMUSG00000081604.4	Gm11518	0.01909	24	14
ENSMUSG00000083899.2	Gm12346	0.01909	24	0
ENSMUSG00000052428.5	Tmco1	0.01909	24	4
ENSMUSG00000027131.3	Emc4	0.01909	24	3
ENSMUSG00000061731.3	Ext1	0.01909	24	4
ENSMUSG00000024370.10	Cdc23	0.01909	24	4
ENSMUSG00000045294.10	Insig1	0.01909	24	2
ENSMUSG00000044221.8	Grsf1	0.01909	24	1
ENSMUSG00000032998.10	Foxj3	0.01909	24	2
ENSMUSG00000071984.4	Fndc1	0.01909	24	4
ENSMUSG00000026585.7	Kifap3	0.01909	24	2
ENSMUSG00000025911.8	Adhfe1	0.01909	24	4
ENSMUSG00000016494.3	Cd34	0.01909	24	2
ENSMUSG00000030298.4	Sec13	0.01909	24	4

ENSMUSG00000022436.9	Sh3bp1	0.01909	24	14
ENSMUSG00000021417.8	Eci2	0.01909	24	4
ENSMUSG00000040928.9	S100pbp	0.01909	24	0
ENSMUSG00000029864.5	Gstk1	0.01909	24	4
ENSMUSG00000001962.8	Fam50a	0.01909	24	21
ENSMUSG00000005078.10	Jkamp	0.01909	24	4
ENSMUSG00000014402.8	Tsg101	0.01909	24	0
ENSMUSG00000031799.9	Tpm4	0.01909	24	4
ENSMUSG00000021756.6	Il6st	0.01909	24	2
ENSMUSG00000000194.7	Gpr107	0.01909	24	2
ENSMUSG00000055866.8	Per2	0.01909	24	22
ENSMUSG00000036940.9	Kdm1a	0.01909	24	2
ENSMUSG00000036099.10	Vezt	0.01909	24	11
ENSMUSG00000034893.7	Cog3	0.01909	24	0
ENSMUSG00000029474.6	Rnf34	0.01909	24	4
ENSMUSG00000024645.4	Timm21	0.01909	24	1
ENSMUSG00000051232.7	Tmem199	0.01909	24	4
ENSMUSG00000029554.9	Mad1l1	0.01909	24	16
ENSMUSG00000066233.5	Tmem42	0.01909	24	2
ENSMUSG00000027217.7	Tspan18	0.01909	24	4
ENSMUSG00000057789.7	Bak1	0.01909	24	12
ENSMUSG00000023992.8	Trem2	0.01909	24	16
ENSMUSG00000036513.9	Commd2	0.01909	24	22
ENSMUSG00000032030.10	Cul5	0.01909	24	4
ENSMUSG00000038936.7	Sccpdh	0.01909	24	2
ENSMUSG00000029759.3	Pon3	0.01909	24	3
ENSMUSG00000043635.6	Adamts3	0.01909	24	4
ENSMUSG00000040158.6	Tax1bp3	0.01909	24	2
ENSMUSG00000001300.9	Efnb2	0.01909	24	4
ENSMUSG00000026271.9	Gpr35	0.01909	24	10
ENSMUSG00000037001.10	Zfp39	0.01909	24	12
ENSMUSG00000049892.7	Rasd1	0.01909	24	4
ENSMUSG00000028369.9	Svep1	0.01909	24	2
ENSMUSG00000020653.5	Klf11	0.01909	24	14
ENSMUSG00000046731.3	Kctd11	0.01909	24	5
ENSMUSG00000035890.8	Rnf126	0.01909	24	8
ENSMUSG00000058729.7	Lin9	0.01909	24	17
ENSMUSG00000015971.4	Actr8	0.01909	24	2
ENSMUSG00000004356.7	Utp20	0.01909	24	0
ENSMUSG00000027313.3	Chac1	0.01909	24	16
ENSMUSG00000026482.7	Rgl1	0.01909	24	2
ENSMUSG00000036995.7	Asap3	0.01909	24	4
ENSMUSG00000086316.1	2210013O21Rik	0.01909	24	4
ENSMUSG00000069378.7	Prdm6	0.01909	24	6
ENSMUSG00000037669.8	1110057K04Rik	0.01909	24	2
ENSMUSG00000031533.3	Mrps31	0.01909	24	4
ENSMUSG00000025507.7	Lrdd	0.01909	24	14
ENSMUSG00000069255.6	Dusp22	0.01909	24	4
ENSMUSG00000030317.6	Timp4	0.01909	24	22
ENSMUSG00000007476.12	Lrrc8a	0.01909	24	4

ENSMUSG00000063889.10	Crem	0.01909	24	2
ENSMUSG00000037296.6	Lsm1	0.01909	24	4
ENSMUSG00000047182.5	Irs3	0.01909	24	4
ENSMUSG00000036819.8	Jmjd4	0.01909	24	11
ENSMUSG00000021339.3	Mrs2	0.01909	24	2
ENSMUSG00000020178.5	Adora2a	0.01909	24	6
ENSMUSG00000004500.8	Zfp324	0.01909	24	2
ENSMUSG00000034748.10	Sirt6	0.01909	24	10
ENSMUSG00000039033.5	Tasp1	0.01909	24	0
ENSMUSG00000033581.10	Igf2bp2	0.01909	24	4
ENSMUSG00000078897.4	Gm4724	0.01909	24	2
ENSMUSG00000007805.3	Twist2	0.01909	24	6
ENSMUSG00000039512.11	Uhrf1bp1	0.01909	24	4
ENSMUSG00000020354.9	Sgcd	0.01909	24	3
ENSMUSG00000097080.1	1700086O06Rik	0.01909	24	12
ENSMUSG00000030935.9	Acsm3	0.01909	24	4
ENSMUSG00000033825.9	Tpsb2	0.01909	24	4
ENSMUSG00000078249.4	Hmga1-rs1	0.01909	24	14
ENSMUSG00000049916.9	2610318N02Rik	0.01909	24	14
ENSMUSG00000053870.6	Fpgt	0.01909	24	4
ENSMUSG00000020151.10	Ptprr	0.01909	24	3
ENSMUSG00000081121.1	Gm12791	0.01909	24	17
ENSMUSG00000003062.8	Stard3nl	0.01909	24	0
ENSMUSG00000025221.9	Kcnip2	0.01909	24	4
ENSMUSG00000022206.6	Npr3	0.01909	24	2
ENSMUSG00000041762.10	Gpr155	0.01909	24	1
ENSMUSG00000057895.5	Zfp105	0.01909	24	1
ENSMUSG00000026048.10	Ercc5	0.01909	24	0
ENSMUSG00000074657.4	Kif5a	0.01909	24	16
ENSMUSG00000090215.2	Trim34b	0.01909	24	15
ENSMUSG00000043943.8	Naalad2	0.01909	24	4
ENSMUSG00000041020.8	Map7d2	0.01909	24	11
ENSMUSG00000050994.13	Adgb	0.01909	24	16
ENSMUSG00000030325.10	Klrb1c	0.01909	24	16
ENSMUSG00000036634.9	Mag	0.01909	24	16
ENSMUSG00000056592.8	Zfp658	0.01909	24	0
ENSMUSG00000028145.7	Them4	0.01909	24	4
ENSMUSG00000027001.4	Dusp19	0.01909	24	3
ENSMUSG00000086877.1	A230072C01Rik	0.01909	24	4
ENSMUSG00000097124.1	A530020G20Rik	0.01909	24	4
ENSMUSG00000078349.2	AW011738	0.01909	24	16
ENSMUSG00000031428.5	Zcchc18	0.01909	24	18
ENSMUSG00000044033.10	Ccdc141	0.01909	24	4
ENSMUSG00000041945.6	Mfsd9	0.01909	24	4
ENSMUSG00000042389.7	Tsen2	0.01909	24	4
ENSMUSG00000011751.10	Sptbn4	0.01909	24	17
ENSMUSG00000032556.9	Bfsp2	0.01909	24	16
ENSMUSG00000012187.7	Mogat1	0.01909	24	4
ENSMUSG00000092124.1	B930094E09Rik	0.01909	24	4
ENSMUSG00000024827.9	Gldc	0.01909	24	21

ENSMUSG00000081895.3	Gm10294		0.01909	24	20
ENSMUSG00000083859.1	Gm12003		0.01909	24	16
ENSMUSG00000059511.3	Gm20563		0.01909	24	4
ENSMUSG00000036095.10	Dgkb		0.01909	24	3
ENSMUSG00000055188.6	2900002K06Rik		0.01909	24	5
ENSMUSG00000087569.1	Gm8464		0.01909	24	16
ENSMUSG00000086670.1	Gm13194		0.01909	24	16
ENSMUSG00000061988.2	Rpl10a-ps2		0.01909	24	16
ENSMUSG00000031297.8	Slc7a3		0.01909	24	1
ENSMUSG00000049832.5	Gm9840		0.01909	24	18
ENSMUSG00000083668.1	Gm5648		0.01909	24	16
ENSMUSG00000035983.4	Gm7008		0.01909	24	16
ENSMUSG00000084941.1	Gm11944		0.01909	24	13
ENSMUSG00000033765.4	Calm4		0.01909	24	22
ENSMUSG00000040035.8	Disp2		0.01909	24	18
ENSMUSG00000034739.11	Mfrp		0.025686	24	4
ENSMUSG00000044948.10	Wdr96		0.025686	24	6
ENSMUSG00000049699.3	Ucn2		0.025686	24	16
ENSMUSG00000085893.1	Gm12091		0.032282	24	12
ENSMUSG00000022389.8	Tef		0.032282	24	21
ENSMUSG00000079037.3	Prnp		0.032282	24	3
ENSMUSG00000081992.1	Gm13408		0.032282	24	16
ENSMUSG00000021794.9	Glud1		0.032282	24	1
ENSMUSG00000030435.9	U2af2		0.032282	24	14
ENSMUSG00000031299.10	Pdha1		0.032282	24	1
ENSMUSG00000030058.11	Copg1		0.032282	24	2
ENSMUSG00000002257.7	Def6		0.032282	24	14
ENSMUSG00000049421.7	Zfp260		0.032282	24	2
ENSMUSG00000034902.11	Pip5k1c		0.032282	24	12
ENSMUSG00000041220.6	Elovl6		0.032282	24	3
ENSMUSG00000021748.8	Pdhb		0.032282	24	2
ENSMUSG00000030245.10	Golt1b		0.032282	24	4
ENSMUSG00000049760.5	2410015M20Rik		0.032282	24	4
ENSMUSG00000039100.9		Mar-06	0.032282	24	2
ENSMUSG00000014444.10	Piezo1		0.032282	24	8
ENSMUSG00000031770.9	Herpud1		0.032282	24	22
ENSMUSG00000022893.8	Adamts1		0.032282	24	2
ENSMUSG00000032116.11	Stt3a		0.032282	24	2
ENSMUSG00000037049.8	Smpd1		0.032282	24	2
ENSMUSG00000054690.11	Emcn		0.032282	24	3
ENSMUSG00000028150.8	Rorc		0.032282	24	2
ENSMUSG00000025511.8	Tspan4		0.032282	24	22
ENSMUSG00000029776.10	Hibadh		0.032282	24	1
ENSMUSG00000032563.9	Mrpl3		0.032282	24	3
ENSMUSG00000090266.4	Mettl23		0.032282	24	3
ENSMUSG00000026077.9	Npas2		0.032282	24	8
ENSMUSG00000025239.2	Limd1		0.032282	24	0
ENSMUSG00000017686.10	Rhot1		0.032282	24	2
ENSMUSG00000021395.10	Spin1		0.032282	24	1
ENSMUSG00000028149.6	Rap1gds1		0.032282	24	1

ENSMUSG00000038991.10	Txndc5	0.032282	24	2
ENSMUSG00000091512.1	Lamtor3	0.032282	24	2
ENSMUSG00000002017.9	Fam98a	0.032282	24	2
ENSMUSG00000032353.7	Tmed3	0.032282	24	2
ENSMUSG00000055319.7	Sec23ip	0.032282	24	0
ENSMUSG00000043252.8	Tmem64	0.032282	24	2
ENSMUSG00000063001.8	Rps23-ps	0.032282	24	0
ENSMUSG00000027367.10	Stard7	0.032282	24	0
ENSMUSG00000034064.8	Poglut1	0.032282	24	4
ENSMUSG00000002210.5	Smg9	0.032282	24	12
ENSMUSG00000095115.1	Itpripl2	0.032282	24	3
ENSMUSG00000016481.10	Cr1l	0.032282	24	0
ENSMUSG00000040374.7	Pex2	0.032282	24	3
ENSMUSG00000002949.8	Timm44	0.032282	24	4
ENSMUSG00000020114.6	Cand1	0.032282	24	0
ENSMUSG00000024269.5	Tpgs2	0.032282	24	3
ENSMUSG00000097971.2	Gm26917	0.032282	24	10
ENSMUSG00000020963.8	Tshr	0.032282	24	4
ENSMUSG00000036334.7	Igsf10	0.032282	24	4
ENSMUSG00000036782.7	Klhl13	0.032282	24	4
ENSMUSG00000001098.9	Kctd10	0.032282	24	2
ENSMUSG00000019874.5	Fabp7	0.032282	24	6
ENSMUSG00000030203.11	Dusp16	0.032282	24	0
ENSMUSG00000059689.8	Zfp637	0.032282	24	4
ENSMUSG00000037613.9	Tnfrsf23	0.032282	24	8
ENSMUSG00000027519.4	Rab22a	0.032282	24	3
ENSMUSG00000024778.6	Fas	0.032282	24	4
ENSMUSG00000035459.9	Stab2	0.032282	24	16
ENSMUSG00000020623.5	Map2k6	0.032282	24	20
ENSMUSG00000032705.8	Exd2	0.032282	24	1
ENSMUSG00000040659.3	Efhd2	0.032282	24	14
ENSMUSG00000048376.5	F2r	0.032282	24	4
ENSMUSG00000036646.7	Man1b1	0.032282	24	0
ENSMUSG00000026608.7	Kctd3	0.032282	24	4
ENSMUSG00000025241.9	Fyco1	0.032282	24	2
ENSMUSG00000025144.11	Stra13	0.032282	24	12
ENSMUSG00000034263.6	Vwa9	0.032282	24	4
ENSMUSG00000034300.10	Fam53c	0.032282	24	1
ENSMUSG00000067369.6	Trmt2b	0.032282	24	0
ENSMUSG00000066150.6	Slc31a1	0.032282	24	3
ENSMUSG00000017724.8	Etv4	0.032282	24	6
ENSMUSG00000028184.8	Lphn2	0.032282	24	5
ENSMUSG00000096173.1	Gm3150	0.032282	24	4
ENSMUSG00000015806.6	Qdpr	0.032282	24	0
ENSMUSG00000029125.8	Stx18	0.032282	24	4
ENSMUSG00000047777.9	Phf13	0.032282	24	2
ENSMUSG00000046532.7	Ar	0.032282	24	0
ENSMUSG00000038764.8	Ptpn3	0.032282	24	0
ENSMUSG00000039476.7	Prrx2	0.032282	24	5
ENSMUSG00000036932.8	Aifm1	0.032282	24	4

ENSMUSG00000052504.6	Epha3	0.032282	24	4
ENSMUSG00000087635.2	Gm13414	0.032282	24	16
ENSMUSG00000037762.6	Slc16a9	0.032282	24	6
ENSMUSG00000005907.8	Pex1	0.032282	24	3
ENSMUSG00000028024.8	Enpep	0.032282	24	8
ENSMUSG00000029815.7	Malsu1	0.032282	24	0
ENSMUSG00000064105.6	Cnnm2	0.032282	24	4
ENSMUSG00000028621.11	Cyb5rl	0.032282	24	4
ENSMUSG00000028152.4	Tspan5	0.032282	24	0
ENSMUSG00000070000.7	Fcho1	0.032282	24	14
ENSMUSG00000038816.8	Ctnnal1	0.032282	24	2
ENSMUSG00000039633.6	Lonrf1	0.032282	24	0
ENSMUSG00000032401.9	Lctl	0.032282	24	4
ENSMUSG00000026810.6	Dpm2	0.032282	24	8
ENSMUSG00000024277.8	Mapre2	0.032282	24	2
ENSMUSG00000036186.5	Fam69b	0.032282	24	4
ENSMUSG00000005225.9	Plekha8	0.032282	24	2
ENSMUSG00000029576.11	Radil	0.032282	24	4
ENSMUSG00000021646.8	Mccc2	0.032282	24	8
ENSMUSG00000024780.6	Cdc37l1	0.032282	24	2
ENSMUSG00000000148.11	Brat1	0.032282	24	4
ENSMUSG00000041406.8	BC055324	0.032282	24	16
ENSMUSG00000042487.5	Leo1	0.032282	24	6
ENSMUSG00000030763.6	Lcmt1	0.032282	24	22
ENSMUSG00000045410.11	Akr1e1	0.032282	24	2
ENSMUSG00000093392.1	Gm6061	0.032282	24	20
ENSMUSG00000029536.7	Gatc	0.032282	24	3
ENSMUSG00000017400.4	Stac2	0.032282	24	12
ENSMUSG00000030722.7	Nfatc2ip	0.032282	24	16
ENSMUSG00000044715.6	Gskip	0.032282	24	4
ENSMUSG00000093548.1	Gm6407	0.032282	24	15
ENSMUSG00000012126.10	Ubxn11	0.032282	24	11
ENSMUSG00000041650.9	Pcca	0.032282	24	1
ENSMUSG00000080875.2	Gm7332	0.032282	24	14
ENSMUSG00000031111.10	Igsf1	0.032282	24	6
ENSMUSG00000089764.1	Gm16580	0.032282	24	16
ENSMUSG00000071291.4	Zfp58	0.032282	24	6
ENSMUSG00000044636.5	Csrnp2	0.032282	24	4
ENSMUSG00000043122.6	A530016L24Rik	0.032282	24	2
ENSMUSG00000095675.1	Ccl21b	0.032282	24	5
ENSMUSG00000040164.3	Kcns1	0.032282	24	5
ENSMUSG00000037463.8	Fbxo27	0.032282	24	2
ENSMUSG00000022371.9	Col14a1	0.032282	24	2
ENSMUSG00000075318.6	Scn2a1	0.032282	24	5
ENSMUSG00000044122.8	Proca1	0.032282	24	2
ENSMUSG00000042505.6	Acn9	0.032282	24	2
ENSMUSG00000014782.9	Plekhg4	0.032282	24	8
ENSMUSG00000085738.2	Gm12335	0.032282	24	0
ENSMUSG00000046561.8	Arsj	0.032282	24	4
ENSMUSG00000028102.9	Pex11b	0.032282	24	2

ENSMUSG00000086679.1	Gm15551	0.032282	24	2
ENSMUSG00000037418.5	Best1	0.032282	24	4
ENSMUSG00000018500.2	Adora2b	0.032282	24	3
ENSMUSG00000091613.1	Gm17046	0.032282	24	4
ENSMUSG00000031482.8	Slc25a15	0.032282	24	0
ENSMUSG00000002083.6	Bbc3	0.032282	24	10
ENSMUSG00000070713.4	Gm10282	0.032282	24	14
ENSMUSG00000020396.8	Nefh	0.032282	24	16
ENSMUSG00000032593.5	Amigo3	0.032282	24	6
ENSMUSG00000047747.9	Rnf150	0.032282	24	20
ENSMUSG00000038583.6	Pln	0.032282	24	4
ENSMUSG00000042401.6	Crtac1	0.032282	24	14
ENSMUSG00000098495.1	RP24-113D21.1	0.032282	24	14
ENSMUSG00000047497.9	Adamts12	0.032282	24	0
ENSMUSG00000057157.3	Gm6054	0.032282	24	4
ENSMUSG00000092454.1	Gm2991	0.032282	24	14
ENSMUSG00000015002.10	Efr3a	0.032282	24	4
ENSMUSG00000028597.11	Gpx7	0.032282	24	2
ENSMUSG00000098198.1	Gm9169	0.032282	24	16
ENSMUSG00000079157.3	Fam155a	0.032282	24	0
ENSMUSG00000020599.7	Rgs9	0.032282	24	4
ENSMUSG00000097554.1	Gm26825	0.032282	24	22
ENSMUSG00000074398.5	Gm15441	0.032282	24	15
ENSMUSG00000095724.1	Gm21319	0.032282	24	20
ENSMUSG00000098985.1	RP24-570C10.6	0.032282	24	19
ENSMUSG00000081540.3	Gm12538	0.032282	24	3
ENSMUSG00000068165.2	Gm10233	0.032282	24	3
ENSMUSG00000005360.8	Slc1a3	0.032282	24	4
ENSMUSG00000062794.8	Zfp599	0.032282	24	12
ENSMUSG00000066270.2	Gm10157	0.032282	24	22
ENSMUSG00000045062.3	Pcdhb7	0.032282	24	6
ENSMUSG00000087433.1	Gm14167	0.032282	24	12
ENSMUSG00000081289.1	Gm14857	0.032282	24	15
ENSMUSG00000047307.1	Pcdhb13	0.032282	24	4
ENSMUSG00000027547.11	Sall4	0.032282	24	17
ENSMUSG00000086884.1	Gm16225	0.032282	24	16
ENSMUSG00000080775.1	Gm6368	0.032282	24	18
ENSMUSG00000033405.3	Nudt15	0.032282	24	3
ENSMUSG00000040904.4	Gm21988	0.032282	24	13
ENSMUSG00000029608.7	Rph3a	0.032282	24	17
ENSMUSG00000003410.7	Elavl3	0.032282	24	16
ENSMUSG00000062257.6	Opcml	0.032282	24	4
ENSMUSG00000035211.8	Xrra1	0.032282	24	6
ENSMUSG00000094248.1	Hist1h2ao	0.032282	24	15
ENSMUSG00000078087.4	Rps12l1	0.032282	24	22
ENSMUSG00000070271.5	Gm13268	0.032282	24	16
ENSMUSG00000062417.5	Hist1h3g	0.032282	24	12
ENSMUSG00000057626.1	Gm5621	0.032282	24	0
ENSMUSG00000082163.1	Gm14276	0.032282	24	12
ENSMUSG00000031995.2	St14	0.032282	24	16

ENSMUSG00000022144.3	Gdnf	0.042414	24	6
ENSMUSG00000030307.7	Slc6a11	0.042414	24	16
ENSMUSG00000009214.3	Tmem8c	0.042414	24	6
ENSMUSG00000034584.3	Exph5	0.042414	24	6
ENSMUSG00000044081.7	4930441O14Rik	0.042414	24	6
ENSMUSG00000036264.9	Fstl4	0.042414	24	18
ENSMUSG00000031376.9	Atp2b3	0.042414	24	5
ENSMUSG00000097760.1	6030442K20Rik	0.042414	24	4
