# Supplementary material for: The intervertebral disc contains intrinsic circadian clocks that are regulated by age and cytokines and linked to degeneration
Source: Ann Rheum Dis. 2017 Feb 17;76(3):576–84. doi: 10.1136/annrheumdis-2016-209428 (PMC5446006; doi:10.1136/annrheumdis-2016-209428)
Supplement: supplementary methods [file annrheumdis-2016-209428supp001.pdf]

Supplementary Online Methods

Animals

All animal studies were performed in accordance with the 1986 UK Home Office Animal Procedures Act. Approval was provided by the local ethics committee. Mice were maintained at 20-22°C, with standard rodent chow available *ad libitum* and under 12:12 hr light dark schedule (light on at 7 am; light off at 7 pm). *Bmal1*$^{flox/flox}$ mice[45] were crossed onto a PER2::luc background. The PER2::Luc mice carry the firefly luciferase gene fused in-frame with the 3' end of the *Per2* gene, creating a fusion protein reporter.[21] *Bmal1*$^{flox/flox}$ - PER2::Luc mice were subsequently crossed with *Col2a1*^{cre} mice expressing cre recombinase under the control of the *Col2a1* promoter[46] to generate cartilage/IVD specific *Bmal1* KO. All mice were bred in-house at the University of Manchester. Genotyping of the *Col2a1-Bmal1*$^{-/-}$ mice was described before.[33]

Reagents and antibodies

IL-1β and TNFα were purchased from R&D, lipopolysaccharides (LPS), BMS-345541, dexamethasone (Dex), Forskolin (FSK) were purchased from Sigma. The following antibodies were used in this study, BMAL1 (mouse monoclonal)[47] and CLOCK (Abcam ab3517).

Tissue explant cultures, bioluminescence recording and imaging

Organotypic IVD tissue explants were prepared as described before.[28] Explants were cultured on 0.4-μm cell culture inserts (Millipore), and bioluminescence was recorded in real time using a LumiCycle apparatus (Actimetrics). Baseline subtraction was carried out using a 24-hour moving average. Amplitude was measured at second peak from the start of recording and period was determined from three peaks using LumiCycle analysis software. For temperature entrainment the incubator housing the LumiCycle after 37°C three day initial phase were set to oscillate the temperature from to 35.5°C for 12 hours to 38.5°C for 12 hours for four cycles. Another incubator was cycling the temperature in opposite phase. Third incubator was kept at constant 37°C temperature as control.

For live tissue bioluminescence imaging, intervertebral discs of the WT and *Col2a1-Bmal1*$^{-/-}$ mice (on a PER2::Luc background) were imaged using a self-contained Olympus Luminoview LV200 microscope (Olympus) and recorded using a cooled Hamamatsu ImageEM C9100-13 EM-CCD camera.[33] Images were taken every hour for the duration of the experiment and combined in ImageJ.

Time course sample collections, mRNA extraction, RNAsequencing and quantitative real-time PCR

The circadian transcriptome studies in mouse IVD were performed as described before.[25] Intervertebral discs were obtained from 8-12 weeks old mice kept under 12 hr/12 hr light/darkness conditions. IVDs were collected every 4 hr for 48 hrs, starting at 9 am (zeitgeber time ZT2). 3-4 lumbar discs of the same animal were pooled to obtain sufficient material. The tissues were immediately snap frozen in liquid nitrogen, and then stored at -80°C until mRNA extraction. Tissues were homogenised using a Mikro-Dismembrator S (Satorius Stedim Biotech) with the barrel and ball of the dismembrator pre-cooled in liquid

nitrogen. mRNA was extracted using RNeasy micro kit (Qiagen) according to the manufacturer's protocol. Quality and integrity of total RNA samples were assessed using a 2100 Bioanalyzer or a 2200 TapeStation (Agilent Technologies) according to the manufacturer's instructions. Thus prepared mRNA was used for RNAseq and qPCR analysis.

For RNA sequencing, RNA-seq libraries were generated using the TruSeq® Stranded mRNA assay (Illumina, Inc.) according to the manufacturer's protocol. Briefly, total RNA (0.1-4 µg) was used as input material from which polyadenylated mRNA was purified using poly-T, oligo-attached, magnetic beads. The mRNA was then fragmented using divalent cations under elevated temperature and then reverse transcribed into first strand cDNA using random primers. Second strand cDNA was then synthesised using DNA Polymerase I and RNase H. Following a single 'A' base addition, adapters were ligated to the cDNA fragments, and the products then purified and enriched by PCR to create the final cDNA library. Adapter indices were used to multiplex libraries, which were pooled prior to cluster generation using a cBot instrument. The loaded flow-cell was then paired-end sequenced (101 + 101 cycles, plus indices) on an Illumina NextSeq instrument. Demultiplexing of the output data (allowing one mismatch) and BCL-to-Fastq conversion was performed with CASAVA 1.8.3. 101bp×101bp paired-end reads were generated from each sample. Up to 82M total reads were obtained in each sample.

For qPCR, RNA concentrations were determined using NanoDrop 2000 (Thermo Scientific), equal amounts of RNA were converted to cDNA using the High Capacity cDNA Reverse Transcription (RT) Kit (Applied Biosystems). Taqman based qPCR was carried out using a StepOne Plus Real-Time PCR System (Applied Biosystems) with Fast Blue qPCR MasterMix (Eurogentec). Taqman primers and probes were purchased from Applied Biosystems. Gene names and probe IDs are as follows, *Gapdh*: Mm99999915_g1; *Arntl*, Mm00500226_m1; *Per2*, Mm00478113_m1; *Dbp*, Mm01194021_m1; *Follistatin*, Mm00514982_m1; *Timp4*, Mm01184417_m1.

Bioinformatic analysis of the RNAseq data

The fastq files were analysed with FastQC and any low quality reads and contaminated barcodes were trimmed with Trimmomactic. All libraries were aligned to GRCm38.p2 assembly of mouse genome using Tophat-2.1.0 and only matches with the best score were reported for each read. The mapped reads were counted by genes with HTSeq against gencode.vM2.annotation.gtf. Genes with very low expressed (with average read across all time point <10) were filtered out. Time-dependent genes were identified by JTKCycle.[29] The rhythmic genes with a 24 hr period and an adjusted p value less than 0.05 were selected for further validation. GO analysis of the JTK_CYCLE identified circadian genes was performed using topGO of the R package.[48] These genes were also clustered with cluster and Rtsne of the R package.[49] Raw data were deposited in EMBL-EBI Array Express (accession number pending).

Human tissues and cells and lentiviral transduction

Adult human IVD specimens were obtained with informed consent from the Intervertebral Disc Tissue Bank at the University of Manchester, using surgical specimens from patients undergoing disc surgery for treatment of disc herniation or IVD degeneration, in accordance

with local ethical committee approval. Tissue was processed for cell extraction and representative samples of all tissues containing intact AF and NP regions were formalin-fixed, paraffin-embedded and sections histologically graded as previously reported[50] with samples graded as follows: non-degenerate (grade 0-4); mildly degenerate (grade 5-7); and severely degenerate (grade 8-12). Only low grade samples (grade 0-2) were used in this study. NP tissue from each sample was macroscopically dissected from AF, and finely minced prior to enzymatic digestion in a solution of 0.1% (w/v) type II collagenase and 0.1% (w/v) hyaluronidase in serum-free DMEM overnight at 37°C with agitation. Isolated cells were cultured in DMEM supplemented with 10% (v/v) FBS, 1 mM sodium pyruvate, 10,000U/ml penicillin, 10mg/ml streptomycin, 25µg/ml amphotericin B and 1mM ascorbate under standard conditions (37°C, 21% O_2, 5% CO_2). Cells were then expanded in monolayer and used at passage <2. Grade 2 cells were used for *Per2::luc* lentiviral transduction and bioluminescence photon counting.

Lentiviral transduction of primary human NP cells was performed using methods previously described.[25] Briefly, lentiviral particles containing a *Per2*::luc reporter were produced in HEK 293T packaging cells and used to transduce the human NP cells. Cells were then synchronized with forskolin before lumicycle recording.

Histology and immunohistochemistry

Mouse spines were dissected and fixed in PBS 4% paraformaldehyde solution followed by decalcification in 20% EDTA pH 7.4. Decalcified tissues were processed and embedded in paraffin. Frontally embedded lumbar spine paraffin blocks were sectioned on a microtome to 5 µm thickness, 3-4 sections per slide. Each block yielded 40-50 slides. Every 5th slide was stained with Saffranin O and the sections were examined for the presence of disc degeneration. H&E, picrosirius red and Safranin O staining were performed according to standard protocols. Safranin O stained sections were imaged using Zeiss Observer D1 Axiocam 105 color camera and measurements of disc height and growth plate thickness were performed in ImageJ. Picrosirius red stained slides were imaged under brightfield or polarized light. The latter allows us to incorporate the birefringent properties of fibrillar collagen, in order to visualize the more organized collagen molecules.

IHC was performed using DAB staining method as described previously.[25] Briefly, the slides were deparaffinised and rehydrated. Antigen retrieval was performed using 1mg/mL Trypsin in PBS (Sigma) digestion for 10 min. Slides were washed and blocked with blocking solution (3% donkey serum in PBS TritonX 0.1%) for 1 h at room temperature. The slides were then incubated with primary antibody diluted in blocking solution overnight at 4°C. Subsequently slides were washed and incubated with fluorescent secondary antibody diluted in blocking solution for 1 h at 4°C. One of the sections on each slide was used as no primary antibody control.

Live imaging of p65-DsRed

Intervertebral discs from p65-DsRed mouse were cut in half to expose both the AF and NP tissue and embedded in the Matrigel matrix (BD Biosciences) in 35-mm glass bottom

Cellview dishes (Greiner Bio-one). Images were acquired with a Zeiss LSM 780 Confocal Inverted Microscope in a humidified CO_2 incubator (at 37°C, 5% CO_2) with a C-Apochromat 40×/1.2 W Korr objective. During imaging tissue was treated with IL-1β (5–20 ng/mL) or TNFα (up to 40 ng/mL). DsRedXP tagged p65 was visualized by excitation with a green helium neon laser (543 nm) and detection through both a 545-nm dichroic mirror and a 560-nm long pass filter. Data capture was performed using ZEN2010B software (Zeiss).

Statistical analysis

Data were evaluated using Two-tailed Student's *t*-test, two way ANOVA or non-parametric, two-tailed Mann-Whitney test. Results were presented as mean ± SEM from at least three independent experiments. Differences were considered significant at the values of $^{*}P < 0.05$, $^{**}P < 0.01$ and $^{***}P < 0.001$.

Supplementary Video 1, Live bioluminescence imaging of the PER2::Luc mouse IVD explants (2 month on the left and 12 month on the right) using high sensitivity EM-CCD camera.

Supplementary Video 2, Live bioluminescence imaging of the PER2::Luc mouse IVD explants treated with IL-1β (at 48 hr), followed by Dex (at 96 hr).

Supplementary Video 3, Live bioluminescence imaging of the mouse IVD explants from a WT mouse (left) and a *Bmal1* cKO mouse (right).
